# The evolutionary signal in metagenome phyletic profiles predicts many gene functions

**DOI:** 10.1186/s40168-018-0506-4

**Published:** 2018-07-10

**Authors:** Vedrana Vidulin, Tomislav Šmuc, Sašo Džeroski, Fran Supek

**Affiliations:** 1 Faculty of Information Studies, 8000 Novo Mesto, Slovenia; 2 Division of Electronics, Rudjer Boskovic Institute, 10000 Zagreb, Croatia; 3 Department of Knowledge Technologies, Jozef Stefan Institute, 1000 Ljubljana, Slovenia; 4 Genome Data Science, Institute for Research in Biomedicine (IRB Barcelona), The Barcelona Institute of Science and Technology, 08028 Barcelona, Spain

**Keywords:** Gene function, Metagenomes, Machine learning, Phyletic profiles, Comparative genomics

## Abstract

**Background:**

The function of many genes is still not known even in model organisms. An increasing availability of microbiome DNA sequencing data provides an opportunity to infer gene function in a systematic manner.

**Results:**

We evaluated if the evolutionary signal contained in metagenome phyletic profiles (MPP) is predictive of a broad array of gene functions. The MPPs are an encoding of environmental DNA sequencing data that consists of relative abundances of gene families across metagenomes. We find that such MPPs can accurately predict 826 Gene Ontology functional categories, while drawing on human gut microbiomes, ocean metagenomes, and DNA sequences from various other engineered and natural environments. Overall, in this task, the MPPs are highly accurate, and moreover they provide coverage for a set of Gene Ontology terms largely complementary to standard phylogenetic profiles, derived from fully sequenced genomes. We also find that metagenomes approximated from taxon relative abundance obtained via 16S rRNA gene sequencing may provide surprisingly useful predictive models. Crucially, the MPPs derived from different types of environments can infer distinct, non-overlapping sets of gene functions and therefore complement each other. Consistently, simulations on > 5000 metagenomes indicate that the amount of data is not in itself critical for maximizing predictive accuracy, while the diversity of sampled environments appears to be the critical factor for obtaining robust models.

**Conclusions:**

In past work, metagenomics has provided invaluable insight into ecology of various habitats, into diversity of microbial life and also into human health and disease mechanisms. We propose that environmental DNA sequencing additionally constitutes a useful tool to predict biological roles of genes, yielding inferences out of reach for existing comparative genomics approaches.

**Electronic supplementary material:**

The online version of this article (10.1186/s40168-018-0506-4) contains supplementary material, which is available to authorized users.

## Background

Many genes still have no known function or have only a very general role assigned. Strikingly, this holds true even for well-studied model organisms*,* where a quarter or more of the genes are poorly characterized [1–3]. Therefore, there is a need to accelerate systematic discovery of gene and protein function using computational approaches for automated function prediction. Large-scale experimental data sets such as protein-protein interactions [4, 5], gene expression measurements [6, 7], and genetic screens [8, 9] have proven valuable for inferring gene function. In addition, genome sequencing enables a complementary set of powerful techniques that are based on comparative genomics. Combining predictions from such bioinformatics techniques with those based on experimental data boosts coverage and accuracy [4, 10–12]. A straightforward and very successful [13] genomic approach is to propagate gene function via homology, which is inferred from gene or protein sequence similarity [14–16].

Furthermore, such annotation transfer by homology is complemented by a variety of “genome context” methodologies which rely on detecting evolutionary patterns across gene families. Prominent examples include analyses of phylogenetic profiles (also called phyletic profiles (PP)), where functional associations between genes are inferred from similar patterns of occurrence of homologs across fully sequenced genomes [17–19]; machine learning can be applied to such data to boost accuracy [20, 21]. Next, conserved gene neighborhoods can also be highly predictive of gene function [22, 23], since neighboring genes are more likely to be co-regulated. An additional genome context approach consists of analyzing evolutionary patterns in codon usage biases [24–26], which serve as a proxy for gene expression levels. Such approaches based on genome data have the advantage of using a pre-existing resource and not requiring costly or time-consuming experimental assays. Of course, targeted follow-up experiments are certainly required for validation of the inferences and to gain mechanistic insight. Genome-based predictors present an opportunity to resolve the functions of many genes, since DNA sequencing is becoming more affordable and thus used to generate vast amounts of data. Simulation studies indicate the accuracy of genome context methodologies stands to profit from such data increases, particularly if diverse methodologies are combined [27].

The number of sequenced whole genomes is steadily increasing, aided by long-read sequencing and assembly [28, 29]. Furthermore, this increase is dwarfed by the amount of data expected from environmental DNA sequencing. A salient example is metagenome sequences describing human-associated microbiota, the focus of much attention because of the promise they hold in preventing and curing disease [30–32]. We reasoned that the sheer abundance of metagenomic data might provide an important opportunity for genome context-based methodologies to predict gene function.

Comparisons between computational function prediction methods indicate that PP are a powerful approach [27, 33]. We thus hypothesized that the PP paradigm might also be fruitfully applied to metagenomic data, yielding an accurate and practically useful methodology to predict gene function. The numbers of metagenomes accessible via public databases are in the thousands, facilitating a systematic evaluation of their utility for this purpose. We employ a simple approach to adapt PP for use on metagenomes, wherein the “metagenome phyletic profile” (MPP) of a gene family consists of its relative abundance across metagenomes. We then apply supervised machine learning [21, 34, 35] to such profiles, finding they are surprisingly accurate in predicting 826 diverse Gene Ontology (GO) terms that describe molecular function, cellular localization, or the biological role of a gene product. Moreover, MPPs provide complementary predictions to standard, whole-genome-based PP, suggesting the utility of MPP as a part of a toolset of contemporary function prediction methodologies. Our analyses also highlight that MPP data derived from metagenomes sampled from different environments is predictive of non-overlapping sets of gene functions. Therefore, increasing the diversity of environments represented in a global metagenomic data set is the key to boosting the ability of MPPs to accurately infer gene function.

## Results

### Metagenome composition can predict the biological roles of gene families

We first examined the general ability of MPPs to predict gene function by constructing data sets using metagenomes sampled from the human gut [36] and from the ocean [37], henceforth referred to as MPP-H (human) and MPP-O (ocean). Here, the data points were 9556 COG or NOG gene families (henceforth collectively referred to as COGs) in MPP-H and 14,331 COGs in MPP-O. The data features were the relative abundances of the COGs in each of the 1267 human-associated (MPP-H) or 139 ocean metagenomes (MPP-O, Additional file 1: Table S1). Similarly to past work using phylogenetic profiling [21, 27, 35], we used the CLUS-HMC supervised machine learning method to predict gene function. This algorithm is based on a Random Forests classifier, adapted to predict multiple outputs (here, GO terms) at once, while improving the accuracy of predictions by drawing on the hierarchical organization of the GO [34, 38]. As described previously [21, 27], we further used cross-validation precision-recall curves to find the precision threshold (*Pr*; also called “positive predictive value”, equivalent to 1-FDR) corresponding to each individual GO prediction. Thereby our methodology provides a probabilistic score for an assignment of each GO term to a COG gene family (see the “Methods” section).

**Fig. 1:**
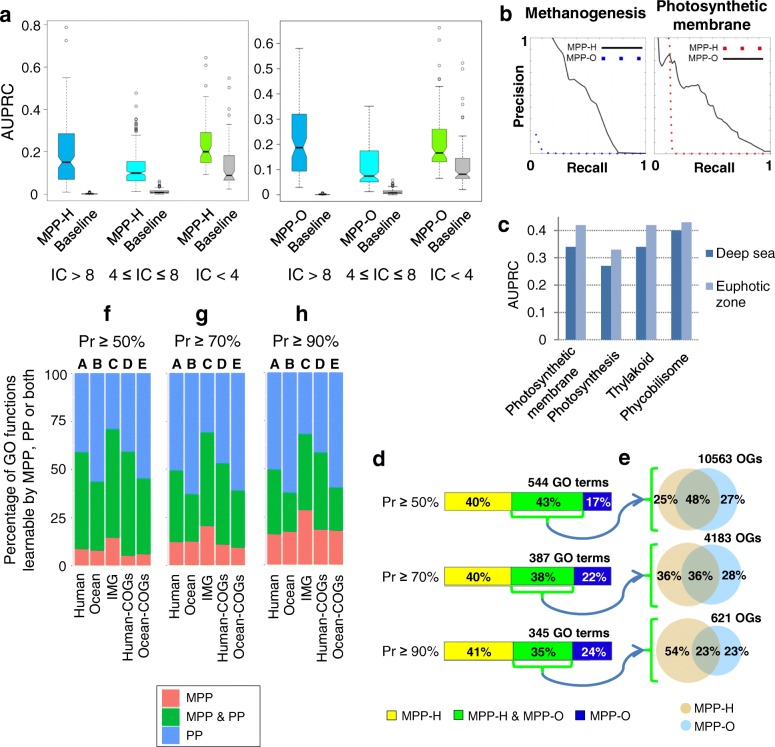
Metagenome phyletic profiling provides novel high-confidence gene function predictions. **a** Distribution of MPP-H and MPP-O model accuracies (expressed as AUPRC score) on 451 and 325 learnable GO functions, respectively. GO functions are stratified according to their frequency of occurrence, expressed as information content (IC). Baseline classifiers are constructed from randomized MPP-H and MPP-O data obtained by randomly reassigning GO functions to COG/NOG gene families. **b** Precision-recall curves for example GO terms learnable only by MPP-H (“methanogenesis”) and only by MPP-O (“photosynthetic membrane”). **c** Predictive accuracies (as AUPRC) for photosynthesis-related gene functions of MPP-O models using deep sea samples and MPP-O models using euphotic zone samples. **d** Overlap between MPP-H and MPP-O inferences, expressed as percent of GO functions predicted only by MPP-H, only by MPP-O or by both. **e** Overlap between MPP-H and MPP-O in terms of particular COGs/NOGs to which only one or both types of MPPs can assign those GO terms that could overallbe predicted by both MPP types (green part of the histogram in (**d**)). **f**–**h** Overlap between different instances of MPP and their matched PPs (Methods), expressed as percent of GO functions predicted only by MPP, only by PP or by both. MPP, metagenome phyletic profiles; PP, phyletic profiles; AUPRC, area under the precision-recall curve

Our analyses indicate that MPP-H data was able to yield at least one prediction at the confidence level of *Pr* ≥ 50% for 451 GO terms, and MPP-O for 325 GO terms. Comparing the two metagenomic data sets, performance was broadly similar both on the highly specific, rarely-occurring GO functions (information content, IC > 8) and the most general, frequently occurring functions (IC < 4; Fig. 1a). We note that a *Pr* threshold of 50% is equivalent to a 128-fold enrichment over random guessing for an example of a highly specific GO term (having IC = 8) and to an eightfold enrichment over random guessing for an example of a general GO term (with IC = 4). Both kinds of MPPs exhibited similar performance across the three GO domains (Additional file 1: Figure S1a). Overall, the “Cellular component” domain was predicted more accurately than the “Biological process” and “Molecular function” GO terms, again consistently for both data sets (Additional file 1: Figure S1a). For all groups of functions, both types of MPPs strongly outperformed the baseline performance, obtained by training a classifier on a randomized data set (*p* < 2 × 10^−16^ for both MPP-H and MPP-O; Mann-Whitney test on AUPRC distribution; Fig. 1a, Additional file 1: Figure S1a). Thus, metagenomes of different environments appear to have overall similar power to predict gene function.

However, we found instances of accurately predicted GO functions that appeared meaningful specifically in the context of the environment represented by a particular MPP. For example, MPP-H but not MPP-O predicts the GO function “Methanogenesis” accurately (cross-validation AUPRC = 0.49 vs 0.02; Fig. 1b). Methanogens are an essential component of intestinal microbial ecosystems, where they promote fermentation of carbohydrate substrates [39]. In contrast to methanogenesis, MPP-O but not MPP-H accurately predicted the GO term “Photosynthetic membrane” (AUPRC = 0.41 vs 0.14, respectively; Fig. 1b). Cyanobacteria, which are a common component of ocean metagenomic samples [37], obtain energy through photosynthesis. MPP-O also successfully predicted several related GO terms including “Photosynthesis” (AUPRC = 0.34), “Thylakoid” (0.41), and “Phycobilisome” (0.47). We further stratified the MPP-O samples by ecological niche in a manner relevant to these examples of photosynthesis-related GO terms and examined the predictive accuracy of our Random Forest models. In particular, cyanobacteria are known to be common in the euphotic zone (up to 200 m of sea depth), which has sufficient sunlight to support photosynthesis [40]. Upon retaining the 29 MPP-O samples extracted from sea depth ≥ 200 m [37], accuracies on the photosynthesis-related functions were lower (cross-validation AUPRC = 0.34, 0.27, 0.34, and 0.40, for GO terms 34357, 15979, 9579, and 30089, respectively) when compared to a random sample of 29 MPP-O metagenomes from the euphotic zone (AUPRC = 0.42, 0.33, 0.42, and 0.43; Fig. 1c).

In summary, the predictive accuracies of classification models derived from human or ocean metagenome data sets are consistently high across the various parts of the GO stratified by domain or by the information content. However, individual GO functions might be more successfully predicted exclusively from metagenomes sampled from particular environments, or from certain niches therein.

### Environmental and human-associated microbiomes predict distinct gene functions

Motivated by the above, we conducted a systematic analysis of the overlap between GO terms “learnable” by the MPP-H versus the MPP-O classifiers, here defined as yielding at least one prediction at an estimated *Pr* ≥ 50%. We found that MPPs constructed from metagenomes representing distinct environments can indeed predict distinct sets of GO functions: only 43% of the GO terms (232 of total 544) are learnable by both MPP-H and MPP-O (Fig. 1d). In other words, of the 544 GO terms learnable by either classifier, 219 can be reliably assigned to at least one gene family only by MPP-H (Fig. 1d) and 93 only by MPP-O. The complementary between the two environments grows even more pronounced at a more stringent threshold of *Pr* ≥ 90%, in which case 142 GO terms are learnable only by MPP-H and 81 by MPP-O, exceeding the number of GO terms (*n* = 122) learnable by both MPPs.

Moreover, we find that even when ocean and human-associated metagenomes can predict the same function, they tend to assign it to a distinct, non-overlapping set of gene families (Fig. 1e). For example, the GO term “Cell motility” can be predicted with similar accuracy by both kinds of MPPs (cross-validation AUPRC = 0.18 and 0.15) and was assigned to 7 and 5 COGs by MPP-H and MPP-O, respectively, of which only 2 COGs overlap. Similarly, “Carbohydrate biosynthetic process” (AUPRC = 0.07 and 0.05) was assigned to 6 and 5 COGs, of which none overlap between MPP-H and MPP-O; all data given at *Pr* ≥ 50%. Of note, the latter example demonstrates how models with an apparently modest AUPRC may in some cases still yield potentially useful predictions, albeit in smaller amounts. Additional file 2 lists AUPRC scores of the predictive models for each GO term and the number of new annotations they yielded at different *Pr* thresholds.

Furthermore, we found cases where a GO term is more accurately inferred by data from one environment, but the second environment may still yield a certain amount of high-confidence predictions that are complementary to the first set of predictions. For example, “Pathogenesis” is more productively learned by MPP-H, yielding 19 COG assignments at *Pr* ≥ 50%, while the MPP-O can annotate only 2 COGs at *Pr* ≥ 50%, which are, however, distinct from the first set. Another example is “Transposition,” assigned to 14 COGs by MPP-O but only 4 by MPP-H, where 3 of those are not covered by the MPP-O results. The individual predictions to gene families for these highlighted functions are provided in Additional file 1: Table S2, while Additional file 3 provides global statistics on the overlap of predicted genes between MPP-H and MPP-O for various GO terms.

Since these examples of metagenomes sampled from two different environments appear to be complementary in terms of gene function annotations they predict, we reasoned that supplementing the MPP-H and MPP-O data sets by additional environments would further boost coverage with confident predictions. We thus introduced metagenomes from the Integrated Microbial Genomes (IMG) database [41]. This larger “MPP-I” data set contains a total of 5049 features (metagenomes) categorized into seven environment groups: freshwater, marine, thermal springs, soil, engineered, human, and plants. These are now considered in addition to the original 1406 metagenomes from the two environments covered by MPP-H and MPP-O. Of note, the following comparisons that include MPP-I are performed on 3536 COGs, without the extended set of NOG gene families absent from the MPP-I data set (see the “Methods” section; we found this yields broadly consistent results as the full set of COG and NOG groups, in terms of the relative coverage of GO terms with accurate predictions; see Fig. 1f–h, columns A vs. D and B vs. E).

### MPPs provide many annotations complementary to standard phyletic profiling

Broadly, the MPP method builds on the idea of PP [17–20]. The standard notion of PP implies examining the similarity in patterns of occurrence of gene homologs across a set of fully sequenced genomes. In contrast, MPP operates on metagenomes, which contain the genetic material of a multitude of organisms that are often not available as individually sequenced genomes (for instance, due to being difficult to grow in pure culture). We thus hypothesized that MPP may be able to predict a distinct set of GO functions, when compared to standard PP.

In order to rigorously test this hypothesis (Fig. 1f–h), we controlled for the possible differences in phylogenetic diversity in the MPP versus PP comparisons. In particular, the full PP data set consisted of 985 microorganisms in which we could map the genes to COGs (see the “Methods” section). However, PP consists of a more diverse set of genomes (total 27 phyla represented, Shannon index (SI; see the “Methods” section) = 1.87, Additional file 1: Figure S1b), while the human gut and the ocean microbiota are less phylogenetically diverse (MPP-H: 4 phyla, SI = 1.2; MPP-O: 36 phyla, SI = 1.55; Additional file 1: Figure S1c, d).

Therefore, we performed experiments to compare matched PP/MPP pairs (Fig. 1f–h), retaining only those microorganisms in PP that belong to phyla present in the environment represented by a particular MPP (see the “Methods” section). Of note, such phylum-based selection is a coarse criterion and the matched PP may still contain individual species that are not present in the MPP. With respect to the number of “learnable” gene functions (defined as above, covered by at least one prediction at *Pr* ≥ 50, 70, or 90%; Fig. 1f–h), we find that metagenomes from MPP-H can reach an additional 5–18% (at different *Pr* thresholds) GO terms that are not learnable by the ordinary PP; for MPP-O, this is 6–18% additional GO terms (Fig. 1f–h, columns D, E). In other words, metagenomic data can help infer gene functions that would not be predicted using only PP constructed from whole genomes. Furthermore, we considered the large MPP-I data set which contains metagenomes from 7 diverse environments. Across different *Pr* thresholds, 15–29% of the learnable GO terms were uniquely reachable only by MPP-I but not by PP (Fig. 1f–h; of note, in this comparison, we use the full PP set, which is appropriate for the diverse MPP-I data set). This proportion is similar to that of the GO terms reachable by PP but not MPP-I, which is 29–32% (Fig. 1f–h).

In summary, a large metagenomic data set that encompasses various environments offers similar predictive power to standard PP, while providing coverage of a complementary set of GO terms. The multi-environment MPP-I data set is superior to the single-environment data sets, suggesting that each individual environment might provide predictions for additional GO terms, which is consistent with the initial MPP-O versus MPP-H comparison (see above). Therefore, we hypothesized that an increase of the number of environments represented will boost coverage of predicted gene functions via MPP; we further test this below.

### A highly diverse set of sampled environments boosts predictive power of MPPs

**Fig. 2:**
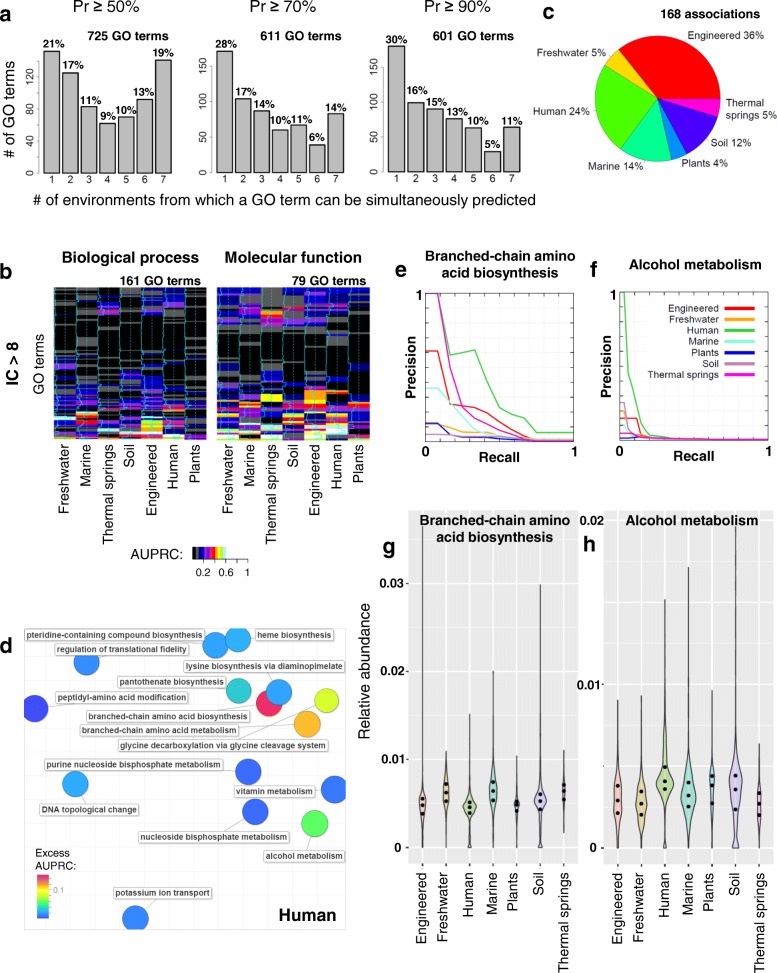
Inferring gene function from metagenomes representing distinct environments. **a** Proportions of Gene Ontology (GO) terms that can be simultaneously predicted from a certain number of environments, expressed for three different stringencies (*Pr* thresholds). **b** Ability to predict GO functions, expressed as the function-specific accuracy of the environment-representing MPP. Rows in heatmaps represent highly specific GO functions (IC > 8), columns are environments, and brighter colors represent higher accuracy (as AUPRC score). Rows are ordered by hierarchical clustering (full dendrogram in Additional file 1: Figure S9). **c** Distribution of the selected associations over seven environment types. **d** A REVIGO plot [82] showing the semantic similarity of the ‘*Biological process*’ GO functions that were associated with the human host metagenomic data. Circle color represents excess accuracy, computed by subtracting the function-specific AUPRC of the second-best MPP from the AUPRC of the best MPP. **e**, **f** Precision-recall curves for two GO functions associated with human host data sets. **g**, **h** Distributions of GO function relative abundances across metagenomes from different environments. Points in the violin plot represent first quartile, median and third quartile. Width of the violin plots is scaled proportionally to the number of observed metagenomes in the group

We could predict many GO functions only from the human gut or from the ocean metagenomes, but not both; see above. Next, we have extended this analysis by individually considering the seven environments that constitute the larger MPP-I data set [41], details in the “Methods” section. Consistently, many of the learnable gene functions could be predicted only from a single environment but not by the remaining six: 21% of GO terms (152 out of 725) received at least one prediction at *Pr* ≥ 50% (Fig. 2a). A further 17% of GO terms were learnable by two of the environments, but not by the remaining five. This trend grows more pronounced at more stringent confidence thresholds: at *Pr* ≥ 90%, 30% of the GO terms are accessible only to a single (of seven) environments in MPP-I (Fig. 2a).

At the same time, some functions were predicted by MPPs of all seven environments (19% GO terms received predictions at *Pr* ≥ 50%, Fig. 2a). We hypothesized that these functions might be related to housekeeping genes, which must be present in most environments. Indeed, we found that 112 of 141 (79%) gene functions predicted by MPPs of all seven environments were housekeeping-related (definition in the “Methods” section [42]), in contrast to only 57 of 152 (38%) GO terms that could be predicted exclusively from a single environment (*p* < 0.0001 by Fisher’s exact test, two-tailed).

Conversely, metagenomes of individual environments contributed specifically to predicting the GO functions of less commonly occurring gene families (Additional file 1: Figure S2a) and also to high information content/rarely occurring gene functions. In particular, the accuracy for a GO function differs considerably among MPP environments for highly specific functions (for GO terms with IC > 8, median of the standard deviations of AUPRC across environments is 0.057; Fig. 2b, Additional file 1: Figure S2b), while it appears to be less variable for general functions (median of standard deviations of AUPRC for GO terms with IC < 4 is 0.008, Additional file 1: Figure S2b).

We further associated gene functions with the specific environments from which they can be successfully predicted. In particular, we consider a GO term to be linked with an environment when the accuracy (cross-validation AUPRC score) of an MPP representing a particular environment is higher than the accuracies of the MPPs representing all other environments for that GO term. As a complementary approach for finding statistical associations, we computed Random Forests feature importance scores (see the “Methods” section), which are commonly applied in human microbiome studies [30–32]. Furthermore, we additionally performed univariate statistical tests to search for associations between GO terms and environments (see the “Methods” section). This yielded 168 robust GO term-environment associations (Additional file 4: Table S3) which were supported by at least two different methods; very general GO functions with IC < 4 were not examined.

More than half of these GO term-environment associations were related to two types of environments (Fig. 2c): (i) the human host, including the digestive, reproductive, respiratory systems and skin, and to (ii) engineered environments e.g. bioreactors, bioremediation sites, waste disposals and wastewaters. Among the former (Fig. 2d), the GO term “branched-chain amino acid (BCAA) biosynthesis” has the highest excess accuracy, when compared to the next best environment (Fig. 2e, AUPRC = 0.37 for human versus 0.22 for thermal springs, respectively). Distribution of the relative abundances of the COG gene families shows that this function is generally depleted in human-inhabiting microbes compared to microbes from the other environments (Fig. 2g; Mann-Whitney FDR = 1.4 × 10^−81^). Therefore, one source of information that was available to the Random Forests to classify a COG as “BCAA biosynthesis” was the low relative abundances of such COGs in humans. A converse example is the relative enrichment of the GO term “alcohol metabolism” in human-associated microbiomes, which may again provide useful signal for predictive models (Fig. 2f, h; AUPRC = 0.09 for human but only 0.03 for engineered environments, FDR = 3.2 × 10^−90^). Turning to the engineered environment metagenomes, we observed a strong association with the GO term “organic phosphonate metabolic process” (Additional file 4: Table S3; AUPRC = 0.87 for engineered environments versus the next best AUPRC = 0.75 for soil, FDR = 1.3 × 10^−14^). Organic phosphonates are used in the manufacture of adhesives, pesticides, and flame retardants and are present in waste disposals [43]. These examples illustrate how gene functions enriched in certain environments provide opportunities for automated function predictions from metagenomes.

### Complementarity of gene functional association networks inferred from MPP and PP

A widely used approach for transferring functional annotations using PP is by constructing gene coevolution networks, where nodes are gene families and edges indicate similarity between the profiles of homolog occurrence across genomes. Following the guilt-by-association principle, the functional annotations are then transferred across the clustered nodes, which have similar profiles [4, 11, 12, 44]. We highlight two examples of the functional association networks constructed from PP versus those constructed from MPP, focusing on prominent instances of GO terms predicted better by either PP or MPP.

**Fig. 3:**
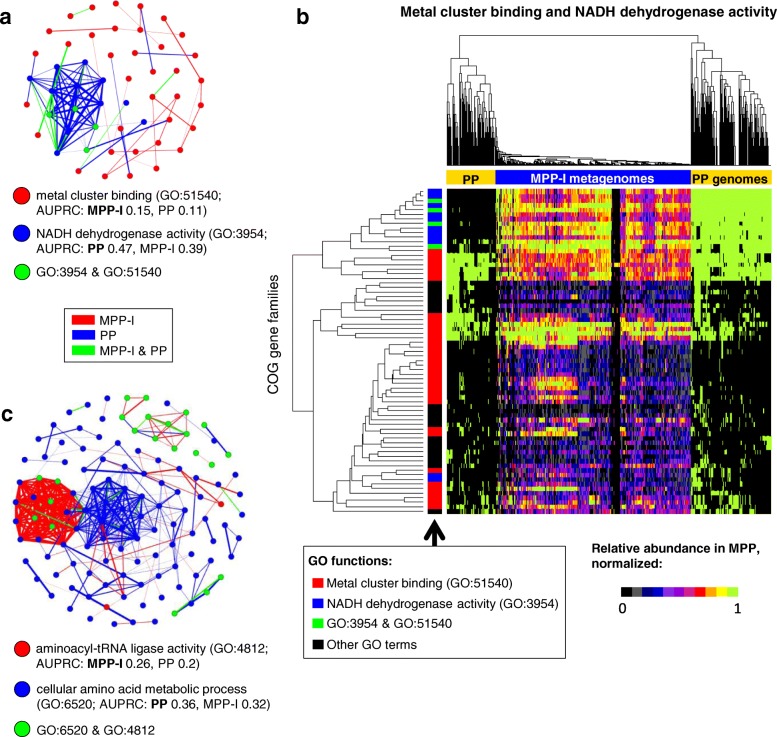
Gene co-evolution networks from metagenome PP and complete genome PP. **a**, **c** Nodes in the network represent COG gene families. Red nodes have assigned the GO function for which MPP-I was more predictive in cross-validation, blue nodes have the GO function where PP was more predictive and green nodes have assigned both functions. A network has two layers: red representing similarity (measured as Pearson correlation) between COG profiles in MPP-I data and blue in PP data. Green edges imply overlap across both layers. Similarities were computed using those metagenomes/genomes that had positive values of Random Forests feature importance (Gini method). **b** The part of MPP-I and PP data sets from which the network in panel **a** is constructed, showing COGs with two selected gene functions from **a**. Additionally, a random selection of negative control COGs that do not have these two functions annotated is shown. Rows are COGs, columns are selected features via Random Forest (complete genomes for PP, metagenomes for MPP), as in panel a. Both the rows and the columns were clustered using complete linkage hierarchical clustering method and Euclidean distance. MPP, metagenome phyletic profiles; PP, phyletic profiles

The first example concerns the function “NADH dehydrogenase activity” (NDA), which was more accurately predicted by PP (matched data set to MPP-I; see the “Methods” section; Additional file 1: Table S1), yielding a cross-validation AUPRC = 0.47 by PP versus 0.39 by MPP-I. Most NDA nodes form a tight cluster via similarity of the PP profiles, but not of the MPP-I profiles (Fig. 3a): all 15 NDA COGs are connected in PP layer versus 7 of them connected in the MPP-I layer (edges represent Pearson *R* > 0.7, corresponding to *p* < 5 × 10^−7^; see the “Methods” section; Additional file 1: Figure S3). In contrast, the GO term “metal cluster binding” (MCB), which partially overlaps NDA (4 of 51 gene families in common), is more accurately predicted by the MPP-I (AUPRC = 0.15) than by the PP (0.11). Consistently, in the coevolution network, a higher number of MCB gene families is connected in the MPP-I layer than it is in the PP layer: 28 versus 21 COGs, respectively, out of 40 COGs connected at Pearson *R* > 0.7. Next, we visualized the PPs of the individual gene families next to their MPPs (Fig. 3b; showing the parts of the profiles found to be informative for gene function via Random Forests feature importance; see the “Methods” section). Upon a hierarchical clustering of the gene families by the pooled PP/MPP data, the COGs with the NDA function are largely separated into a cluster characterized by pattern evident in the PPs but not the MPPs (see top of the heatmap in Fig. 3b). In turn, the COGs with the MCB function are well-separated from a random sample of COGs having neither of the two functions, where the pattern evident in the MPPs forms a basis for this clustering (Fig. 3b).

The second example is the function “cellular amino acid metabolic process” (CAAM), which was slightly more accurately predicted by Random Forests trained on PP (AUPRC = 0.36) than on MPP-I (0.32), while a partially overlapping function “aminoacyl-tRNA ligase activity” (ATLA) was better predicted by MPP-I (AUPRC = 0.26) than by PP (0.2). Consistently, many of the CAAM nodes are interconnected in a cluster reflecting a high similarity of PP: 84 out of 118 in the PP layer, compared to 75 in the MPP-I layer of the network. However, for ATLA (where 22 of 24 nodes also have CAAM assigned), such gene families have more interconnections in MPP-I than they have in the PP network layer (21 versus 10, respectively, out of 24; Fig. 3c), consistent with higher Random Forest performance observed with MPPs. These examples illustrate the differential signal in gene coevolution networks derived from PPs or MPPs that can be captured by machine learning models to systematically assign many different functions to genes via the PPs and via the MPPs.

### Taxon relative abundance data can provide accurate function prediction models

Above, we have demonstrated how metagenomic data can be used to predict gene function. However, compared to metagenomes, a more abundant source of environmental DNA data comes from sequencing of the 16S rRNA gene. This enables a quantification of the relative abundance of microbial taxa, but does not provide information on abundance of individual genes. However, gene-level information can be approximated from 16S rRNA data using tools such as PICRUSt [45] or Tax4Fun [46], which have proven sufficiently accurate to provide biological insight [45]. Given that 16S data is less costly to obtain and therefore prevalent compared to metagenomes, we asked if drawing on this data source can provide useful gene function predictions. To this end, we collected 20,570 16S rRNA gene samples representing distinct environments (Additional file 1: Table S4) from the Qiita database [47] and approximated metagenome composition using PICRUSt v.1, which draws on a built-in set of 2590 genome sequences, which are then combined by weighting by the 16S rRNA-derived taxon relative abundance (see the “Methods” section). Predictive power of these 16S rRNA gene-based MPP (MPP-16S) was compared against the accuracy of the whole metagenome MPP-I data set. We took a random sample of 5049 MPP-16S to provide a balanced comparison to MPP-I in terms of number of features, and measured cross-validation AUPRC scores across 3536 COGs and 3358 GO terms assigned to them.

**Fig. 4:**
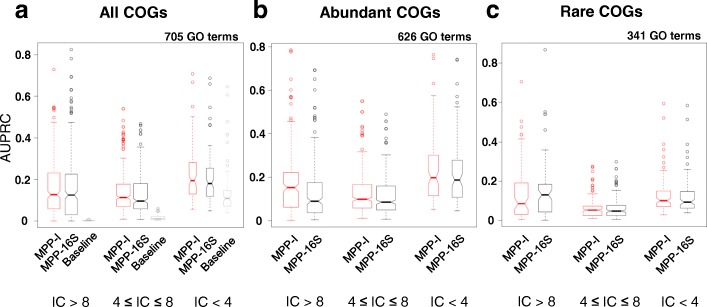
Metagenome composition approximated from taxon abundance obtained via 16S rRNA gene sequencing can predict gene function. **a**–**c** Distribution of Random Forest classifier accuracies on learnable GO terms, separated according to the subset of COGs used to construct the classifier and according to GO term generality levels (as information content, IC). Box plot width represents the proportion of binned GO terms

Interestingly, the MPP-16S appear to be highly predictive of gene function, approaching the predictive performance of the metagenome-based MPP (Fig. 4a); both methods perform significantly better than a randomized baseline (AUPRC_MPP-*I*_ = 0.16 ± 0.12 versus AUPRC_16S_ = 0.15 ± 0.13, mean ± standard deviation; both have *p* < 10^−15^ by Mann-Whitney test on AUPRC distribution versus baseline, which has AUPRC_baseline_ = 0.03 ± 0.06). The predictive power of MPP-16S compares favorably in particular for the highly specific gene functions (Fig. 4a, Mann-Whitney test *p* = 0.88, indicating there is no difference in location of AUPRC distributions between MPP-16S and MPP-I, across the set of GO categories with IC > 8).

We hypothesized that the reason why MPP-16S performs comparably to ordinary (metagenome) MPP-I may have to do with low sequencing coverage of rare genes in metagenomes, which would result in a noisy abundance readout in MPP-I. The accuracy on the most specific (highest IC) functions would be most affected by such noise in metagenome MPP-I, since the machine learning algorithm relies on a small number of training examples (COGs) to learn them. To test the above hypothesis, we divided the COGs into abundant (above-median relative abundance in metagenomes; see the “Methods” section) and rare, and constructed separate classification models to predict gene function for both groups. In the case of abundant gene families, the MPP-I performs significantly better than MPP-16S on the specific functions with IC > 8 (Fig. 4b, *p* = 2 × 10^−4^ by Mann-Whitney test; AUPRC_MPP-*I*_ = 0.18 ± 0.16 versus AUPRC_16S_ = 0.14 ± 0.14). In contrast, this MPP advantage over MPP-16S is reversed on rare gene families (Fig. 4c; *p* = 0.04, AUPRC_MPP-*I*_ = 0.14 ± 0.14 versus AUPRC_16S_ = 0.15 ± 0.15). We interpret this as the MPP constructed from 16S rRNA metagenomic data being able to compensate for the inevitable inaccuracy of the computationally estimated gene family relative abundances [45] by providing more precise estimates for rare genes than the direct readouts from metagenome sequencing, which are accurate but may be imprecise. Moreover, the available 16S rRNA gene data sets are currently more numerous than metagenomes and are available for very diverse environments (our set listed in Additional file 1: Table S4), which works in favor of MPP-16S.

### Validating the metagenomic function predictions using independent experimental data

While computational inferences are useful in helping elucidate functions of poorly characterized genes, the predictions need to be confirmed by experiments. We therefore examined how many of our MPP annotations can be validated using the data from Critical Assessment of Functional Annotation 2 (CAFA2) [1], a community effort at benchmarking gene function prediction methods. In brief, the CAFA2 data set consists of experimentally determined gene function annotations that accumulated in public databases during a specific time period (here, Jan 2014 to Sep 2014), which can then be used to evaluate the predictive models trained only on data available prior to the initial time point.

Our training data meets this requirement (see the “Methods” section), and we can therefore use the CAFA2 data for independent validation. The majority of CAFA2 data points for prokaryotes were given for *Escherichia coli* and *Pseudomonas aeruginosa*, and we therefore evaluated our predictions from MPP-I on these two bacteria. At *Pr* ≥ 50%, MPP-I assigned 64 validated GO functions to 39 (out of total 70) unannotated *E. coli* genes that were covered by CAFA2. Complementarity of methods was evident in that 22 of these 64 GO terms were assigned to at least one gene to which the matched PP did not provide the same prediction (Additional file 5: Table S5). Furthermore, MPP-I assigned a notable amount of validated annotations also at the more stringent *Pr* ≥ 70%: 41 GO functions to 28 (of 70 CAFA2-supplied) genes. Similar trends are observed for *P. aeruginosa*, where MPP-I assigned 90 validated GO functions to 40 genes (out of 53 CAFA2-supplied) at *Pr* ≥ 50%, where the majority of them (68 of 90) were assigned to at least one gene to which PP did not give the same prediction (Additional file 5: Table S5). Therefore, MPP models could uniquely predict function for tens of genes that validated in subsequent experimental data, when considering two well-investigated microbes. When comparing the accuracy of the PP and MPP-I classifiers presented herein to a broader set of methods participating in the CAFA2 challenge, both the PP and MPP-I range between the 1st and the 2nd quartile of the distribution by the F-max measure in various tests (Additional file 1: Figure S4). This suggests overall rather accurate methods with potential to contribute to the combination methods that tend to be the top-performers in the CAFA challenges [1, 27, 48–50].

**Fig. 5:**
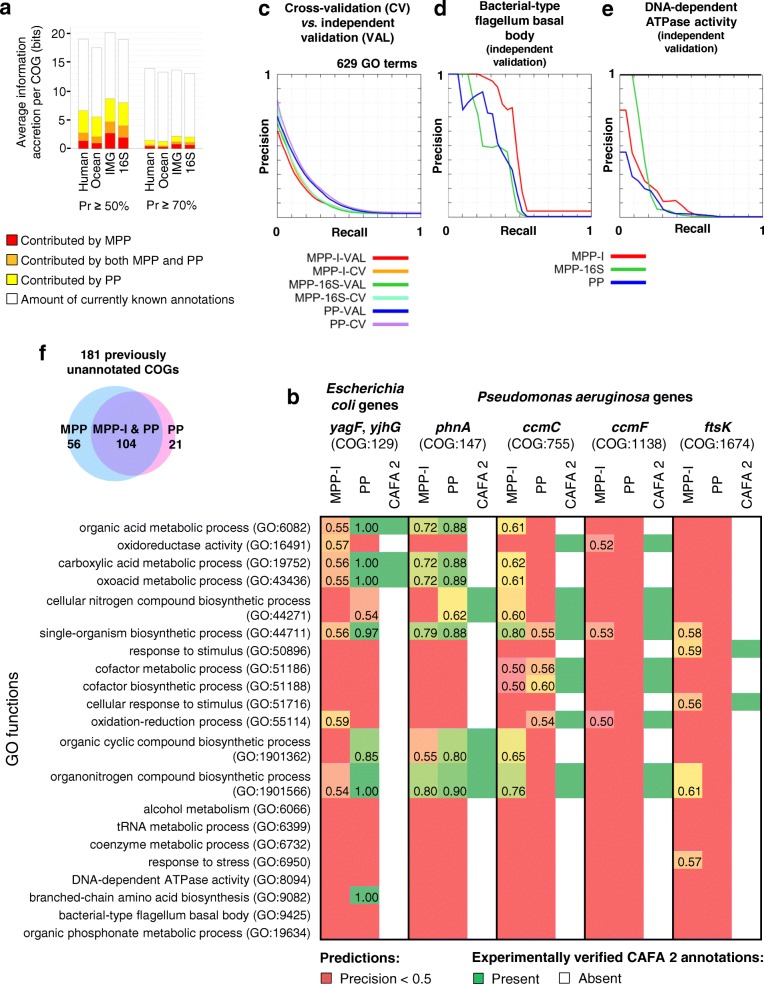
Validation of gene function inferences made by MPP on independent data sets. **a** Average information accretion, per COG, of the novel gene function annotations assigned by MPP, by PP or by both, versus information per COG in the currently known annotations. **b** Examples of annotations validated in the *E. coli* and *P. aeruginosa* CAFA2 benchmark data. Columns represent precision (*Pr*) scores assigned by MPP-I, *Pr* scores assigned by PP and confirmed predictions on CAFA2 for six example genes from either *E. coli* or *P. aeruginosa* genes. Rows are GO functions. Red color in MPP-I and PP columns indicates that a classifier did not predict a GO function at *Pr* ≥ 50%. The complete set of predictions is given in Additional file 6: Table S5. Bottom part of heatmap shows a sample of gene functions that did not receive CAFA2 annotations in the shown gene. **c** Precision-recall curves represent average data over individual curves for 629 GO terms. VAL, validation; CV, cross-validation. **d**, **e** Precision-recall curves for individual GO terms computed from an independent validation set of most recent UniProt-GOA annotations. **f** Proportions of unannotated COGs that received at least one new Uniprot-GOA validated annotation by MPP-I, by PP or by both

We turn to examine examples of individual CAFA2-validated predictions (Fig. 5b). MPP-I, for instance, assigned an annotation to the *P. aeruginosa ccmC* gene, predicting it to have the function “organonitrogen compound biosynthesis” at *Pr* = 76%. This was not predicted by PP at *Pr* > 50%, but was validated in the CAFA2 set (Fig. 5b). Similarly, the *ftsK* gene was correctly predicted by MPP-I but not PP to have the function “response to stimulus” at *Pr* = 59%. A contrasting example is provided by the *phnA* gene, where the correct function “organic cyclic compound biosynthesis” was predicted more confidently by the PP than the MPP-I model (*Pr* = 80% and 55%, respectively). Overall, these examples (Fig. 5b) illustrate the complementary nature of the MPP-derived and the PP-derived predictions. Of note, we also predicted many gene functions that were absent in the CAFA2 prokaryotic data set (Additional file 6 provides gene-level predictions for nine selected microbes). Since absence of an annotation in the CAFA2 set does not imply absence of function, it is currently difficult to quantify to what extent such predictions correspond to false positives or to *bona fide* discoveries.

### Validating the estimates of model accuracy on a large external set of function annotations

The prokaryotic part of the CAFA2 set contains high-confidence experimental function annotations, which limits its scope. In addition, many additional data have accumulated since CAFA2 ending time point (Sep-2014), up to the date of our analyses. We therefore collected a larger independent set of annotations by using an up-to-date version of the Uniprot-GOA database [51] from a wide range of organisms and including various types of evidence for assigning functions, including computational annotations. This validation set encompasses 1941 COGs and 629 GO terms (details in the “Methods” section). Importantly, similar to the CAFA2 set above, it uses only annotations newer than the ones used for constructing our classifiers (up to Dec-2013) and is thus independent from our training data examples. The large size allowed us to systematically test accuracy of the predictive models.

Encouragingly, the accuracy of MPP-I and MPP-16S measured on the validation set is rather consistent with the original estimates from cross-validation that were also used to determine *Pr *scores for each prediction (Fig. 5c; MPP-I: AUPRC = 0.118 ± 0.110 (mean ± standard deviation) vs. 0.136 ± 0.112 for independent validation data and cross-validation on original data, respectively; MPP-16S: 0.129 ± 0.112 vs. 0.138 ± 0.113; matched PP: 0.158 ± 0.128 vs. 0.175 ± 0.132). The independent data set also supports the notion that MPPs provide added value over standard PPs for a number of gene functions: for example, MPP-I predicted the term “bacterial-type flagellum basal body” with accuracy higher than PP: AUPRC = 0.475 vs. 0.345 for MPP-I and PP, respectively, on the independent data set; Fig. 5d. Similarly, both MPP-16S and MPP-I were more successful than PP in predicting the GO term “DNA-dependent ATPase activity”: AUPRC = 0.176 (16S), 0.136 (MPP-I) vs. 0.087 (PP; Fig. 5e).

Furthermore, this independent validation set suggests that—compared to PP—MPP-I models appear to be particularly good at predicting function for currently fully unannotated gene families, which are arguably of more interest for applying function prediction methods. Of the 309 unannotated COGs (without any known GO function) that were present in the independent validation set, MPP-I and PP together annotated 181 COGs at *Pr* ≥ 50%: 56 out of 181 received validated annotations exclusively by MPP-I, while 21 of 181 received validated annotations solely by PP (Fig. 5d). This provides evidence that MPPs are a valuable new addition to the toolbox of automated function prediction methods because they yield a complementary set of predictions for gene families without a known function.

### MPPs elucidate a substantial amount of novel information about microbial gene function

We next compared MPP to PP in terms of the amount of novel functional annotations they can assign to COG gene families. This includes those COGs which did not previously have any known function assigned to them (see the “Methods” section) and also the COGs with known functions for which we predicted additional novel functions. Each predicted annotation was weighted with a measure of the amount of information it contributes to the knowledge of gene function, here quantified by the information accretion (IA) measure [52]. IA is expressed in bits and tends to be higher for rarely occurring functions (details in the “Methods” section).

When examining predictions with *Pr* ≥ 50%, the most highly predictive IMG data set yielded 2.6 bits/COG that could be predicted exclusively by the MPP-I but not by the matched PP representation, while 4.0 bits/COG were contributed solely by PP. At a more stringent threshold of *Pr* ≥ 70%, these relative contributions were upheld, with 0.7 bits/COG provided only by MPP-I, and 0.9 bits/COG only by the PP (Fig. 5a). Therefore, a substantial amount of novel predicted gene functions is assigned exclusively by MPP and not by PP. The converse also holds, implying that the approaches are best used in combination.

In addition, these results suggest that the larger metagenomic data set (MPP-I) yields a higher amount of novel gene function information compared to the smaller MPP-H and MPP-O (shown in Fig. 5a). However, this observation is confounded by the higher diversity of sampled environments in MPP-I compared to H and O which both consist of samples from more uniform environments. This prompted us to systematically examine the individual influence of data set size (in terms of numbers of metagenomes) and diversity (in terms of number of different environments represented) via simulation studies.

### Diversity but not quantity of metagenomes determines the accuracy of MPP models

**Fig. 6:**
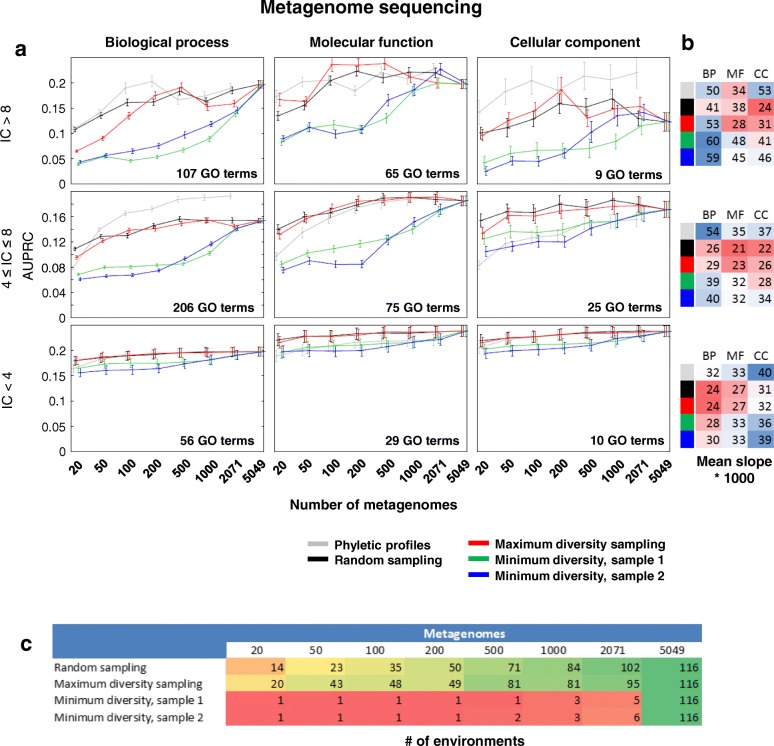
Increasing diversity rather than amount of metagenomes is crucial for accurate gene function prediction. **a** *x*-axes represent the number of sampled metagenomes. *y*-axes represent cross-validation AUPRC averaged over GO functions from a specific GO domain (column) and of a specific level of generality (row; stratified by IC). Error bars represent standard error of the mean. Maximum diversity sampling approximately retains the proportions of samples from the environments represented in the full data set. Minimum diversity sampling always begins with the largest environment (e.g., soil); in the second experiment ("sample 2") in **a** all samples representing soil were removed from the data and the sampling was started from the second largest environment. **b** slopes of the linear regression fits of accuracy scores against increasing data set size for the PP and MPP data sets (using different sampling approaches), showing the average slope of all segments connecting neighboring points in plot; complete data in Additional file 1: Table S6. **c** The number of environments contained in each data set. BP, Biological process; MF, Molecular function; CC, Cellular component; IC, Information content

The standard phyletic profiling approach has been shown to increase in accuracy as the number of available genomes increases, but with diminishing returns [2, 27]. This suggests that possible benefits might still be reaped by increasing the number of genomes past the last tested point (*n* = 2071 in reference [27]), but also that the increase would need to be substantial to be practically useful. Here, we tested this using metagenomes and MPPs, which provide an abundant source of genomic data with a tendency to grow very fast in the future. For the three GO domains and various generality levels of GO terms, the largest current set of metagenomes (*n* = 5049) does not outperform smaller metagenome sets (Fig. 6). Overall, a set of ~ 2000 randomly sampled features (metagenomes) was very similarly predictive as the full 5049 feature set (average AUPRC for *n* = 2071 is 0.174 ± 0.125 and for *n* = 5049 is 0.173 ± 0.124; mean ± standard deviation). We further evaluate the metagenomes approximated from 16S rRNA gene relative abundance data using PICRUSt [45]. Again, a random sample of ~ 2000 features is only modestly less predictive than the full set (AUPRC for *n* = 2071 is 0.180 ± 0.133 and for *n* = 20,570 is 0.190 ± 0.130). Therefore, past approximately 2000 metagenomes, the increases in accuracy are nearly negligible, given one important consideration: that a random sampling of the currently available data is used. Such sampling conserves the representation of diverse environments in the reduced-size data sets.

Our previous analyses of complementarity of the predicted GO functions between different environments (Fig. 2) suggest that increasing the diversity of the data set—the number of environments it spans—could have an important effect on predictive accuracy. To systematically test this, we use two sampling strategies: MaxD, which maximizes the diversity of environments within the sample, and MinD, which minimizes the diversity by sampling only from a single environment until all its metagenomes are exhausted, then moving onto the next environment (details in Methods). Remarkably, the MaxD strategy achieves near-maximum accuracy with only *n* = 200 metagenomes (average AUPRC = 0.169 ± 0.132 versus 0.173 ± 0.124 for the full *n* = 5049 metagenome set; Fig. 6). In contrast, the MinD strategy with *n* = 200 samples only from a single environment and reaches much lower scores (average AUPRC = 0.100 ± 0.097 and 0.095 ± 0.100 for two independent runs of MinD, which sample only from the soil and human oral microbiome environments, respectively; Fig. 6). As a control, we have examined the effect of reducing redundancy within the MPP-I data set by removing correlated MPPs, while ignoring the diversity of environment labels. This yields no systematic gain of accuracy at moderate stringency (Pearson *R* ≤ 0.9, *n* = 1039 metagenomes remaining) and a loss at a higher stringency (*R* ≤ 0.7, *n* = 412 metagenomes remaining) (Additional file 1: Figure S5), suggesting that the removal of redundant features by itself does not benefit predictive power in our experimental setup, thereby highlighting the importance of environment-specific signal for accurately predicting gene function.

Repeating the diversity analyses using PICRUSt-approximated metagenomes obtained from 16S rRNA gene relative abundance data yields a similar result: MaxD sampling with only *n* = 200 provides accuracy (AUPRC = 0.171 ± 0.121) closer to the maximum with the very large set of *n* = 20,570 features (0.190 ± 0.130) than is the case for MinD (0.130 ± 0.125) (Additional file 1: Figure S6). Overall, these analyses demonstrate how diverse metagenomes, rather than simply large numbers of metagenomes, are required to obtain accurate models for computational function prediction.

## Discussion and conclusions

Our work suggests that environmental DNA sequencing provides a rich source of data for predicting gene function in a systematic, unbiased manner. In particular, we adapted phylogenetic profiling, a well-established method for detecting gene functional associations [17–19] to draw on metagenomic data and accurately predict GO terms by using a machine learning methodology derived from Random Forests [34, 35, 38]. In our MPP pipeline, individual metagenomes are used in place of individual fully sequenced genomes (as in PP), and metagenomic relative abundance of gene families is used instead of presence/absence patterns of gene homologs (as in PP). Strikingly, this rather straightforward approach is similarly predictive of gene function as are whole-genome PP, while—crucially—yielding a very large number of complementary inferences (Figs. 1 and 4). Metagenomes sampled from different environments are predictive of distinct, non-overlapping sets of gene functions (Figs. 1, 2 and 3). Consistently, the diversity of environments present in the pooled set of currently available MPPs determines the total predictive power of the MPP-based approaches. We note an analogy to past work that has proposed phylogenetic diversity to be beneficial to predictive accuracy of the classical PPs, derived from individual genomes [53–57].

The amount of metagenomic data is rapidly increasing, which has the potential to benefit the MPP-based automated function prediction pipelines in the future. This however depends on the kind of metagenomes that will be made available: our simulations (Fig. 6) suggest that, for instance, the inevitable arrival of many additional human gut microbiome sequences will likely not considerably improve the MPP's ability to infer microbial gene function. In contrast, we predict that the addition of more exotic metagenomes, such as those from extreme environments, from specialized bioreactors or from bioremediation sites would be very revealing of functions of poorly characterized gene families. We foresee several directions for future research related to the MPP methodology.

Firstly, it is important to learn about how to integrate the predictions made by MPP with those of other methods, including those drawing on large-scale experimental data as well as on comparative genomics [11, 12, 58–60]. Our recent work suggests that genome-based function predictors, including PP, might be best integrated by a (perhaps counter-intuitive) strategy of simply trusting a single highly-confident call even when it is not supported by multiple methods [27]. By analogy, refining the strategies for data integration may result in tangible benefits for the practical use of MPP, depending on how their constituent environments and sub-environments are treated when training global predictive models. Secondly, the MPP approach may also be useful for the determining gene function for eukaryotic and viral constituents of metagenomes. Recent developments have adapted the PP methodology to eukaryotic genomes by accounting for the evolutionary history of the involved species and the duplication events within individual gene families [61, 62]. A conceptually similar approach might apply to MPP of eukaryotic genes. Thirdly, an important consideration that concerns all methods is whether the predictions are sufficiently trustworthy to be useful for prioritizing for experimental follow-up. Existing function prediction pipelines commonly provide confidence scores in arbitrary units, which reflect relative ranks but are difficultto interpret otherwise. Here, we used cross-validation and precision-recall curves to provide FDR estimates for each prediction, an approach we previously found to be broadly accurate when predicting gene function [21] and also microbial phenotypes [63]. Still, benchmarking the algorithms on external data sets is invaluable, and systematic efforts to do so via community challenges for function prediction methods are gaining traction [1, 64]. Fourth, an extension of the MPP methodology could in principle be used to predict function also for genes that are observed in metagenomes, but that cannot be confidently assigned to the existing COG/NOG (or similar) gene families via sequence similarity. Gene families defined using genome sequences of organisms grown in pure culture may not adequately capture the vast genetic diversity of the currently unculturable microbes, which is evident in metagenome sequencing.

This study provides an example of how metagenomes can be used to derive phylogenetic profiles that are useful for automated prediction of Gene Ontology terms. Future work is needed to investigate whether, in addition to PP, other comparative genomics methods could also successfully draw on metagenomic data. An example of this has been proposed, which is based on the conserved gene neighborhoods approach. In particular, putative operons can be inferred from neighboring genes in the same metagenomic DNA sequencing read, in cases where the read is sufficiently long and well-positioned to span multiple genes. Then, the guilt-by-association principle can be applied to infer function of poorly characterized genes that reside in the same segments with well-described genes [65–67]. Future improvementsin running costs and in error rates of long-read technologies will likely increase the utility of this “proximon” approach. A further opportunity may lie in the methodologies to infer gene function [24, 26] and phenotype [25, 63] from the evolution of codon usage biases, a proxy for gene expression levels in a variety of living organisms [68, 69]. Codon biases appear to be consistent within metagenomes and are also predictive of expression levels in metaproteomes [70], providing a rationale for using codon biases to infer gene function from metagenomes at a large scale.

In conclusion, environmental DNA sequencing has provided a toolkit for deepening our understanding of free-living and human-associated microbial communities. We suggest that metagenomes additionally constitute a general tool for systematically inferring gene function.

## Methods

### Metagenome phyletic profile and phyletic profile data sets

The human gut microbiome MPP (MPP-H) data set is composed of 1267 microbiomes/features, 9556 eggNOG v3 [71] COG and NOG groups (training instances) and 3886 GO terms/labels. Feature values represent the sum of COG/NOG member genes’ relative abundances retrieved from the Integrated reference catalog of the human gut microbiome [36].

The ocean microbiome MPP (MPP-O) data set is composed of 139 metagenomes and 14,331 OGs COGs and NOGs labeled with 4087 GO terms. Feature values were retrieved from the Ocean microbial reference catalog [37].

The integrated Microbial Genomes (IMG) MPP (MPP-I) data set is composed of 5049 metagenomes and 3536 COGs labeled with 3358 GO terms. Feature values were computed from the data downloaded from the IMG database [41] in April 2016.

The phyletic profiles (PP) data set is composed of 985 bacterial and archaeal genomes/features and 15,575 eggNOG v3 COG/NOGs labeled with 4213 GO terms. Feature values represent COG member genes’ presence/absence throughout 985 complete genomes. The data for constructing PP was downloaded from NCBI (ftp://ftp.ncbi.nih.gov/genomes/) folders Bacteria and ASSEMBLY_BACTERIA in October, 2014. The data set includes the genomes that could be mapped to the eggNOG v3 COG/NOGs, and the COG/NOGs that are present in ≥ 5 of the 985 genomes.

### Subsets of PP matched with various MPP by phylum composition

PP matched with MPP-H (PP-H) contains a subset of 765 genomes from PP belonging to the four phyla reported in human gut microbiome data (Additional file 1: Figure S1c, [36]). PP-H has 9556 COG and NOGs (in the text collectively referred to as COGs) labeled with 3886 GO terms. To make balanced comparisons between MPP-H and PP-H, we retained in both data sets only those COGs that overlap between MPP-H and PP.

PP matched with MPP-O (PP-O) contains a subset of 139 genomes from PP belonging to the phyla present in more than 1% of detected microorganisms (Additional file 1: Figure S1d, [37]). More specifically, we sampled genomes in the same proportions of phyla as they appear in the 139 metagenomes. Comparisons with MPP-O were based on the common set of 14,331 COGs labeled with 4087 GO terms.

PP matched with MPP-I (PP-I) is composed of 2071 genomes and 3536 COGs labeled with 3358 GO terms. Considering that MPP-I is composed only of COGs (not NOGs) and that COGs are matched between eggNOG v3 and v4, we used information from the eggNOG v4 database [72] to map genomes to COGs.

MPP-I contains only COGs, and in order to make fair comparisons with MPP-H and MPP-O, we constructed MPP-H-COGs and MPP-O-COGs. MPP-H-COGs is composed of the same set of metagenomes as MPP-H, but 3568 COGs labeled with 3404 GO terms instead of the full set of COGs and NOGs. Similarly, MPP-O-COGs have the same set of metagenomes as MPP-O, but 3699 COGs labeled with 3420 GO terms. Matched versions of PPs were constructed from PP-H and PP-O with the matching number of COGs.

### Assigning gene ontology functional annotations to COGs/NOGs

In all data sets, a COG gene family was annotated with a set of GO terms that were originally assigned to ≥ 50% of COG member genes, counting only across genes that initially had any GO term assigned (Additional file 7). Annotations with evidence codes denoting both the experimental and the electronic annotations from all three GO domains were assigned to COGs, while propagating upwards to the GO root. GO was downloaded from Uniprot-GOA database [73] from December 2013. We investigated to what extent our subsequent analyses are robust to this “≥ 50% genes” heuristic for propagating gene function across the member genes of a COG by also testing a more stringent threshold (≥ 70% genes) and a more permissive one (≥ 30% genes in a COG must have function assigned). We found this has no substantial effects on accuracy of the models nor on the complementarity between predictions provided by the MPP and standard PP, which remains pronounced (Additional file 1: Figure S7).

In the analysis, we differentiate GO terms by their *generality*, which is expressed though Shannon Information Content (IC) that assigns high scores to infrequently used terms [74]:

$$ \mathrm{IC}\left({GO}_{\mathrm{i}}\right)=-{\log}_2\mathrm{frequency}\left({GO}_{\mathrm{i}}\right) $$

IC was measured among UniProt-GOA genes of the 2071 genomes that received at least one annotation.

Phylogenetic diversity was measured using Shannon index [75]:

$$ SI=\sum \limits_{i=1}^P{p}_i\ln {p}_i $$

where *p*_i_ is the proportion of phylum *i*.

### Hierarchical multi-label classification

Classification models were constructed using CLUS-HMC [76] with default parameters, except for these settings: decision tree pre-pruning to prevent the algorithm to form a leaf node when the number of instances in the node is < 5; forests size to 200 trees; size of a feature subset for Random Forests to square root of the total number of features. Predictions were collected for annotated (from the out-of-bag cross-validation procedure) and in some experiments also for unannotated COGs. For each COG, a classifier outputted a vector of confidence scores ranging from zero to one, which indicate classifier’s confidences in assigning each of the GO terms to the COG.

### Converting confidence scores into precision (*Pr*) scores

The confidence scores for classification models were converted into *Pr* scores which, unlike the confidences, have a probabilistic interpretation: they are equivalent to 1-false discovery rate. First, for each model, the mapping between confidences and *Pr* scores were computed separately for each GO term by constructing a precision-recall (P-R) curve. In particular, this entails: varying confidence thresholds from 1.0 to 0.0, with the step of 0.001, consequently increasing the number of COGs annotated with the GO; computing at each threshold true positives (TP) that represent the number of correctly predicted true annotations, false positives (FP) that represent the number of incorrectly predicted true annotations and *Pr* score that represent a proportion of predictions known to be true: TP/(TP + FP). Then, for each COG-GO pair, confidence score was rounded to three decimals and substituted with *Pr* score related to that specific confidence threshold and the GO of interest. All predictions having *Pr* scores ≥ 0.1 for various types of MPPs and matching PPs are in Additional file 8.

### Evaluation measures in cross-validation

Classification models performance in cross-validation (out-of-bag procedure [77]) was evaluated using P-R curves and the Area under the P-R curve (AUPRC) scores. P-R curves were computed separately for each GO term by varying a Pr threshold from one to zero and collecting at each threshold TP, FP, false negatives (FN) that represent the number of missed true annotations, precision (TP/(TP + FP)) and recall that represents a proportion of true annotations that were successfully predicted (TP/(TP + FN)). Intermediate P-R points were estimated using linear interpolation. In some cases, GO-specific P-R curves were averaged. We presented P-R curves on a graph where recall is on *x* and precision on *y*-axis. AUPRC was computed as area enclosed between *x*-axis and a curve (it should be noted that when min. observed recall was > 0, the precision computed at this minimum point was estimated at recall = 0 point in order to close the curve). The more the curve is shifted to the right (AUPRC closer to one), the better the model performance is. In addition to using out-of-bag error estimates, we also tested the complementarity of the MPP and PP methods using five-fold cross-validation, which provided broadly similar results in terms of MPP being able to provide many additional predictions not accessible to PP and vice versa (Additional file 1: Figure S8).

### Extraction of environment-specific functions

Metagenomes from MPP-I were divided into seven data sets based on the environment from which they were sampled: freshwater (690), marine (846), thermal spring (191), soil (977), engineered (580), human-associated (876), and plant-associated (230) metagenomes. The environments were selected from the top three levels of the environment-representing tree provided by the IMG database. All seven data sets have a common set of instances, which are 3536 COGs with at least one of the 3358 GO functions assigned. From each data set, a classification model was constructed with CLUS-HMC.

A GO function was associated with an environment based on three function-related statistics computed for each environment: cross-validation AUPRC (from environment-specific classification model), Random Forests feature importance (RFFI, details below), and false discovery rate (FDR) from Mann-Whitney statistical test (details below). For each function, we first selected the environment for which AUPRC was higher than the AUPRCs for all the other environments. We repeated this procedure for the other two statistics. Finally, we extracted a set of robust associations for which AUPRC-selected environment matched with at least one of the RFFI- or FDR-selected environments. RFFI- and FDR-based selection are approaches frequently used in the related work [30–32].

RFFIs were computed from the seven data sets, one for each environment. In all data sets, features were 725 GO functions with at least one correct prediction at Pr ≥ 50% outputted by the environment-specific classifiers, instances were 4390 metagenomes representing the environments, and feature values were sums of relative abundances of function-associated COGs. Data sets differed in associated class values, which indicated whether metagenomes were sampled from that specific environment. From each data set, a classifier was constructed with FastRandomForest [78] using default parameters, with an exception of the size of the forests, which was set to 500. In this implementation, RFFI represent a reduction in classifier’s accuracy after feature values randomization.

FDRs were computed from the same seven data sets used to compute FIs. For each environment, i.e., data set, *p* values were computed for 725 GO functions by performing the Mann-Whitney statistical test using each GO function’s relative abundances in 4390 metagenomes and the binary indicator of whether metagenomes were sampled from that particular environment. Computed *p* values were then FDR-adjusted using Benjamini-Hochberg.

GO terms related to a set of housekeeping genes were obtained by matching gene identifiers from the list of housekeeping genes in [42] (Additional file 1: Table S2) to the gene identifiers in the Uniprot-GOA database using gene ID cross-references [79], and collecting GO terms related to the matched genes.

### Gene co-evolution networks

A separate network was constructed for a pair of GO functions. Nodes in the network are COGs with these two functions assigned in the Uniprot-GOA. The network has two layers, one representing similarities between COG profiles in the MPP-I data set and the other representing similarities between COG profiles in the MPP-I matched PP data set. Before computing similarities, a feature selection step was performed based on the Random Forests feature importances obtained using the “randomForest” R package (200 trees, random seed of one). For this purpose, MPP-I data set was assigned with a binary class that represents the presence of a GO function on which MPP-I showed better performance compared to PP. In the case of PP, binary class showed the presence of GO function where PP performed better. Feature importances were measured as a total decrease in node impurities from splitting on a feature, averaged over all trees. The node impurity was measured as Gini index. We kept the features with positive values of Gini. Similarity was then measured using Pearson correlation coefficient (*r*) on the reduced number of features. We considered absolute values of *r* and omitted the edges with *r* < 0.7. The threshold for *r* was selected at a point in *r* distributions (Additional file 1: Figure S3) that leaves a manageable number of edges in all of the presented networks. Thickness of edges represents the value of *r* and in case that the two layers overlap, the thickness represents an average between *r* computed from MPP-I and PP profiles. We kept in the network only those nodes that have at least one edge.

In Fig. 3b, MPP relative abundances were normalized to the same scale as PP, meaning they were scaled to range between zero and one. More specifically, for a set of MPP relative abundances *x* = (*x*_1_,...,*x*_*n*_), a normalized value *y*_*i*_ was obtained for each relative abundance value *x*_*i*_ by applying the formula:

$$ {y}_i=\frac{x_i-\min (x)}{\max (x)-\min (x)} $$

### 16S rRNA gene abundance analyses

16S rRNA gene-based MPP (MPP-16S) is constructed from operational taxonomic unit (OTU) abundance tables downloaded from Qiita database [47]. We collected 20,570 samples from 64 studies covering various environments (Additional file 1: Table S4). OTU tables were input into PICRUSt v.1 [45] to construct COG abundance profiles. Abundances were then normalized to range between 0 and 1 within each sample, as described above for MPP. The resulting tables were merged into a single data set by retaining information for 3536 COGs common to MPP-I. Finally, for a fair comparison with MPP-I, we randomly extracted 5049 out of 20,570 samples.

When splitting each of the MPP-16S and MPP-I data sets into two representing abundant and rare COGs, we considered as abundant those COGs that have median relative abundance in the upper 50% of distribution of all data set COGs, and rare otherwise.

Classification models were constructed with CLUS-HMC. Accuracy was measured as AUPRC, and in the analysis, we retained only learnable functions for which the two MPPs provided at least one prediction at Pr ≥ 50% measured in cross-validation.

### Validation of novel functional annotations

Novel annotations are those that were assigned by MPP or PP to COGs and were not previously associated with those COGs based on the Uniprot-GOA database from December, 2013 (used for training the classifiers, Additional file 7). This means that for COGs that were used for training the classifiers, the predictions were extracted from the cross-validation. We also considered annotations assigned to COGs without any known annotation. In the case of MPP-H we considered novel annotations for 3568 COGs already in MPP-H and additional 742 unannotated COGs; MPP-O: 3699 and 815; MPP-I and MPP-16S: 3536 and 1095. In the case of PP, PP-H and PP-O have matched number of COGs with MPP-H and MPP-O, but in the case of PP-I and PP-16S there is a matched number of annotated COGs, while the number of unannotated COGs is 635. It should be noted that in this analysis, we considered only COGs in MPP-H and MPP-O (and their matched PPs) to be able to make fair comparisons between different instances of MPPs.

Annotations were weighted using information accretion (IA), which assigns high scores to GO terms that contribute with new information when added as a specialization of a parent or a set of parent terms [52]:

$$ \mathrm{IA}\left({\mathrm{GO}}_i\right)=-{\log}_2P\left({\mathrm{GO}}_i|T\right) $$

*T* is a set of parent terms in GO and P denotes conditional probability.

IA was computed using the SemDist R package [80] among UniProt-GOA genes of the 2071 genomes that received at least one annotation.

We downloaded CAFA2 benchmark from [81]. The majority of annotations for prokaryotes were available for *E. coli* and *P. aeruginosa* including 70 *E. coli* “no-knowledge” benchmark genes (with no previous annotations in all three domains) and 53 *P. aeruginosa* genes with associated experimentally verified annotations. On this benchmark, we validated annotations predicted by the MPP and PP classifiers constructed from the training sets annotated with GO terms downloaded from Uniprot-GOA database dated December 2013 to meet the requirement of the CAFA2 challenge.

To form the second, broader validation set, we downloaded all GO annotations from the Uniprot-GOA database in November 2016 (Additional file 7) and removed GO annotations that were available before December 2013 (this is the original set of annotations used throughout our work, Additional file 7). Additional file 9 provides the number of COGs assigned to GO terms, given the known annotations from Uniprot-GOA versions December 2013 or November 2016. In summary, we obtained 1941 COGs (of the 3536 in the full MPP-I data set) that had received at least one new GO term during the period December 2013 to November 2016. Out of the GO terms newly assigned to the 1941 COGs, we selected the 629 GO terms that were also deemed “learnable” (received at least one prediction at *Pr* ≥ 50%) by either MPP-I, MPP-16S, or from the matched PPs and proceeded with evaluation on that set of GO terms.

### Influence of the number and diversity of metagenomes on MPP accuracy

Simulations were performed using MPP-I (5049 metagenomes) and MPP-16S (20,570 16S rRNA gene microbiomes) data by applying three types of sampling: besides random sampling, we applied two diversity-based sampling strategies that use information on distribution of metagenomes/16S rRNA microbiomes over environments. Maximum diversity sampling aims to retain the same ratio of metagenomes from the environments represented in the data set. In contrast, minimum diversity sampling first uses all of the metagenomes from the largest environment, then from the second largest and so on.

We associated MPP-I metagenomes with 116 environments from the fourth level of the environment-representing tree provided by the IMG database. In comparison, environments in Fig. 2 were taken from the top three levels. Examples of environments are: Environmental -> Terrestrial -> Soil -> Loam, Host-associated -> Plants -> Rhizoplane -> Epiphytes, Engineered -> Wastewater -> Industrial wastewater -> Petrochemical.

In the case of the 16S rRNA gene abundance data, microbiomes were associated with 89 environments. We considered that one study equals one environment with the exception of the following four studies: “Alaskan arctic tundra ecosystem” study was divided into 7 environments: biofilm (247), freshwater (2595), freshwater sediment (145), marine (24), marine sediment (32), soil (105), and unclassified (5). “Bacterial communities associated with different human sites” study was divided into 7 environments: gut (45), hair (14), nose (46), oral (46), skin (357), urine (48), and unclassified (44). “Human microbiome” study was divided into 3 environments: skin (992), oral (508), and gut (467). “Microbes in Melbourne water catchments” study was divided into 12 environments according to an animal from which a fecal sample was found beside a water catchment: cat (4), dingo (1), dog (32), emu (7), fox (24), goose (29), kangaroo (477), possum (8), rabbit (263), sambar deer (943), wombat (178), and unclassified (28).

## Additional files

Additional file 1: The document contains **Tables S1,** **S2,** **S4** and **S6**, and **Figures S1-S9**. (PDF 1680 kb)

Additional file 2: Accuracy of MPP-H, MPP-O, and MPP-I classification models, as the AUPRC score (using out-of-bag estimates provided by Random Forest) for individual GO terms. Next to each AUPRC score, the table lists the number of COG/NOG gene families for which that GO term is predicted at *Pr* thresholds of ≥ 10% (least stringent), ≥ 30%, ≥ 50%, ≥ 70% and ≥ 90% (most stringent). (XLSX 298 kb)

Additional file 3: Statistics of overlap between MPP-H and MPP-O predictions made for genes of nine example microbes, tallying assignments of GO terms at confidence of *Pr* ≥ 50% for the set of GO terms that can be simultaneously predicted by both MPP-H and MPP-O. These statistics reflect only predictions that match known annotations in the Uniprot-GOA database version Dec-2013. (ZIP 18 kb)

Additional file 4: **Table S3.** Associations between 725 GO terms and 7 environments. Each row in the table represents an association. The 168 associations highlighted in the text are at the top of the table and the selected environment is given in the “EA-Environment” column. (XLSX 117 kb)

Additional file 5: **Table S5.** MPP-I vs. matched PP predictions that validated on CAFA 2 *Escherichia coli* and *Pseudomonas aeruginosa* benchmarks. Tables contain annotations with precision scores *Pr* ≥ 0.5 assigned by MPP-I and their matched PP to benchmark genes and the experimentally verified annotations from the benchmarks. Rows are clustered using the R package *Seriation* (column “Order”). (XLSX 152 kb)

Additional file 6: MPP-I, MPP-H and MPP-O predictions for genes of nine example microbes, given in separate directories in the archive, which are named after the NCBI Taxonomy ID of the selected strain. Two files in each directory contain predictions at a permissive threshold of Pr ≥ 0.1, where one file contains all predictions and the other only predictions that correspond to previously known annotations in the Uniprot-GOA database (December 2013). In the files, columns represent: gene name or synonym, COG/NOG or COGs/NOGs mapped to the gene, and the remaining columns are GO terms (header contains GO ID) that had received at least one prediction at *Pr* ≥ 0.1 (other GO terms are omitted from the table); cells contain the exact Pr scores at which each prediction was made. (7Z 6989 kb)

Additional file 7: Known GO annotations downloaded from the Uniprot-GOA database of (i) December 2013 and assigned to eggNOG v3 COG/NOGs, (ii) December 2013 and assigned to eggNOG v4 COG/NOGs, and (iii) November 2016 and assigned to eggNOG v4 COG/NOGs; in all cases using the 50% rule for propagating GO functions across genes within a COG or a NOG. (7Z 391 kb)

Additional file 8: Gene family function annotations predicted by MPP-H, by MPP-O, by MPP-I, by MPP-16S, by PP (matched with MPP-H), by PP (matched with MPP-O) and by PP (matched with MPP-I and MPP-16S), all provided in separate table files. In each table, rows are gene families (first column lists the COG or NOG ID), columns are GO functions (the header row lists GO ID) and values are the precision (*Pr*) thresholds at which a GO term was assigned to a COG or a NOG. Values of *Pr* < 0.1 are listed as *Pr* = 0. Table contains the set of GO terms with at least one prediction available at the threshold Pr ≥ 0.1. (7Z 18097 kb)

Additional file 9: Number of COG and NOG gene families that are assigned to GO terms, considering the known GO function annotations from Uniprot-GOA dated either December 2013 or November 2016, and for COGs/NOGs from either the eggNOG database v3 or eggNOG v4. (XLSX 140 kb)

## Abbreviations

AUPRC: Area under the precision-recall curve
CAFA: Critical Assessment of Function Annotation
COG: Cluster of Orthologous Groups
FP: False positive
GO: Gene Ontology
IC: Information content
MPP: Metagenome phyletic profile
PP: Phyletic profile (or phylogenetic profile)
Pr: Precision
TP: True positive
