# Supplementary material for: The evolutionary signal in metagenome phyletic profiles predicts many gene functions
Source: Microbiome. 2018 Jul 10;6:129. doi: 10.1186/s40168-018-0506-4 (PMC6040064; doi:10.1186/s40168-018-0506-4)
Supplement: Supplementary file 1 — The document contains Tables S1, S2, S4 and S6, and Figures S1-S9. (PDF 1680 kb) [file 40168_2018_506_MOESM1_ESM.pdf]

Automated gene function prediction using metagenome data

Vedrana Vidulin, Tomislav Šmuc, Sašo Džeroski, Fran Supek.

Additional file 1: Supplementary tables and figures

Table S1. Description of metagenome phyletic profile (MPP) data sets and their matched phyletic profile (PP) data sets. COG, cluster of orthologous genes. NOG, non-supervised orthologous groups. GO, Gene Ontology.

MPP			Matched PP	Common characteristics		
	Metagenomes	Environments	Genomes	COG/ NOGs	GO terms	Shared phyla
MPP-H	1267	Human gut microbiome	765	9556	3886	4 – Fig. S1c
MPP-O	139	Ocean microbiome	139	14331	4087	7 – Fig. S1d >=1%
MPP-I	5049	Freshwater, marine, thermal springs, soil, engineered, human, plants from the IMG database	2071	3536	3358	PP is composed of all available fully sequenced genomes
MPP-16S	20570	16S rRNA samples from environments in Table S4	2071	3536	3358	

Table S2. Example sets of gene families to which only MPP-H, only MPP-O or both models assigned a specific GO term. Abbreviations as in Table S1.

GO term	MPP-H	MPP-H & MPP-O	MPP-O
Carbohydrate biosynthetic process (GO:0016051)	COG763, COG774, COG1212, COG1560, COG1663, COG3563	-	COG381, COG1044, COG1083, COG1091, COG1898
Cell motility (GO:0048870)	COG1256, COG1291, COG1536, COG1558, NOG42595	COG1815, COG1868	COG1677, COG1749, COG2063
Pathogenesis (GO:0009405)	COG5613, NOG11699, NOG12853, NOG13696, NOG14612, NOG18563, NOG25967, NOG25973, NOG26011, NOG40012, NOG40270, NOG42629, NOG43838, NOG46381, NOG47700, NOG71760, NOG74835, NOG85163, NOG149123	-	NOG14341, NOG149417
Transposition (GO:0032196)	COG3436, NOG28899, NOG261425	COG3547	COG2963, COG3039, COG3293, COG3328, COG3385, COG3464, COG3666, COG4644, COG5421, COG5433, NOG4436, NOG44148, NOG122322

Table S4. The individual studies representing distinct environments that were sampled from the Qiita database. A number of samples represents a subset of samples from a study for which precomputed operational taxonomic units (OTUs) are available.

Environment/Study	Study ID	# samples
Amazonian leaf microbiome	10245	120
Antibiotic perturbation of the murine gut microbiome	10469	391
Alaskan arctic tundra ecosystem	1883	3153
Bacterial communities associated with the lichen symbiosis	929	16
Bacterial communities associated with the surfaces of fresh fruits and vegetables	1671	214
Bacterial communities present on fermented foods	10395	32
Bacterial community on eggshells	1694	562
Barn swallow microbiome	231	83
Bat fecal microbiome	1734	94
Beach sand microbiome	10145	114
Bee microbiome	1064	387
Bird gut microbiome	1773	122
Bovine milk bacterial communities	10485	228
Cannabis soil microbiome	1001	26
Caporaso Glen Canyon soil microbiome	1526	95
Chick gut microbiome	10291	119
Chu Changbai mountain soil microbiome	1702	22
Co-digestion microbiome	10137	183
Bacterial communities associated with different human sites	449	600
Disordered microbial communities in the upper respiratory tract of cigarette smokers	524	290
Estuarine bacterioplanktonic communities	10470	128
Florida decay wastewater microbiome	1818	198
Golden frog bacterial community	10196	37
Green iguana hindgut microbiome	963	100
Gut microbiome of hibernating bears	2300	96
Gut microbiota in Burmese pythons	391	130
Gut microbiota of Grants gazelles	10323	745
Gut microbiota of wild ring-tailed lemurs	10407	44
Hawaii Kohana Volcanic soil microbiome	1579	128
Human microbiome	550	1967
Hydra microbiota	1364	39
Infant fecal samples	10293	130
Intestinal microbes of sleep deprived flies	1799	154
Kakamenga Kenya soil microbiome	1711	77
Kilauea geothermal soils microbiome and biofilms	895	5
Lung microbiome of HIV infected individuals	959	143
Malaysia Lambir soil microbiome	1713	34
Mammalian corpse decomposition microbes	10142	635
Marine mammal skin microbes	1665	186
Microbes in Melbourne water catchments	894	1994
Microbes from public restroom surfaces	1335	109
Microbial communities of whitehead bats gut	2338	192
Microbial communities on money	375	660
Microbial flora in ant-eating mammals	1056	93
Microbiology of malting and brewing	10105	499
Microbiota of freshwater fish slime and gut	940	275
Microbiota of Ixodes ticks	1885	139
Microbiota of the insect gut	10124	32
Microorganisms from cold polluted coastal sediments	1198	61
Mongolian steppe microbes	864	230
New Zealand terrestrial Antarctic microbes	1035	121
Nicaragua coffee soil microbiome	1715	61
Gut microbiome in obese and lean twins	77	281
North Atlantic water column microbiome	2080	54
Oral microbiota in captive Komodo Dragons	1747	210
Gut bacteria of Peruvian rainforest ants	10343	471
Microbiome of green roofs in New York	1674	151
Bacterial communities associated with river sediment particles	807	44
Metagenome of soil at different pH levels	805	14
Microbes associated with the bulk soil, rhizosphere, roots, leaves, flowers and grapes from 4 Merlot vine clonal varieties	1024	348
Sponge microbiome	1740	1403
Squirrel gut microbiota	926	46
Zebrafish intestinal microbiota	1192	47
Whitehead fish microbiome	10308	1208

Table S6. Slopes of the lines from Fig. 6a and Fig. S6a. Values in the table represent slopes of the regression lines for phyletic profiles and metagenome phyletic profiles with different sampling approaches. Large numbers (green boxes) represent steeper slopes, which indicate larger improvements in accuracy (measured as cross-validation area under precision-recall curve (AUPRC)) with addition of new metagenomes. In contrast, small numbers (red boxes) represent less steep and negative slopes, which indicate saturation and suggest no further improvement from additional metagenomes. Slopes for (**a**) metagenomes from MPP-I and (**b**) 16S rRNA from MPP-16S. Abbreviations: IC = information content.

Metagenome sequencing

(a) Biological process

IC		≤ 20	≤ 50	≤ 100	≤ 200	≤ 500	≤ 1000	≤ 2071	≤ 5049
IC > 8	Phyletic profiles	83	81	165	43	-91	24	48	
	Random sampling	83	70	85	2	54	-66	68	30
	Maximum diversity sampling	50	65	147	134	40	-126	18	98
	Minimum diversity, sample 1	30	39	-27	25	34	73	159	147
	Minimum diversity, sample 2	33	33	29	35	54	69	87	132
4 ≤ IC ≤ 8	Phyletic profiles	60	153	85	23	38	10	7	
	Random sampling	84	50	4	52	26	-7	0	-1
	Maximum diversity sampling	73	67	55	8	21	17	-31	23
	Minimum diversity, sample 1	52	29	2	9	5	56	120	35
	Minimum diversity, sample 2	47	13	6	23	47	77	80	30
IC < 4	Phyletic profiles	126	45	26	6	9	8	2	
	Random sampling	138	14	13	8	9	6	-1	2
	Maximum diversity sampling	139	17	10	12	3	0	6	1
	Minimum diversity, sample 1	126	22	5	-2	6	12	32	18
	Minimum diversity, sample 2	120	11	3	8	24	29	27	18

(a) Molecular function

IC		≤ 20	≤ 50	≤ 100	≤ 200	≤ 500	≤ 1000	≤ 2071	≤ 5049
IC > 8	Phyletic profiles	126	70	9	-65	86	-40	55	
	Random sampling	98	50	154	58	-31	36	-3	-56
	Maximum diversity sampling	121	-8	230	-3	7	-84	-34	-6
	Minimum diversity, sample 1	60	65	22	-26	51	167	50	-6
	Minimum diversity, sample 2	65	54	-42	29	137	66	120	-72
4 ≤ IC ≤ 8	Phyletic profiles	52	67	51	39	16	10	9	
	Random sampling	76	34	16	29	19	3	-7	-3
	Maximum diversity sampling	71	43	37	29	2	11	2	-11
	Minimum diversity, sample 1	46	31	17	17	15	34	63	31
	Minimum diversity, sample 2	41	26	-14	1	68	66	44	25
IC < 4	Phyletic profiles	141	30	25	23	6	3	5	
	Random sampling	166	17	2	14	3	-1	7	5
	Maximum diversity sampling	162	29	-1	8	16	-2	2	5
	Minimum diversity, sample 1	147	26	8	6	9	5	29	34
	Minimum diversity, sample 2	148	5	-2	4	19	30	15	42

(a) Cellular component

IC		≤ 20	≤ 50	≤ 100	≤ 200	≤ 500	≤ 1000	≤ 2071	≤ 5049
IC > 8	Phyletic profiles	131	123	86	-75	85	-29	52	
	Random sampling	93	33	68	121	-21	66	-149	-20
	Maximum diversity sampling	89	89	79	161	-168	72	20	-95
	Minimum diversity, sample 1	39	56	25	3	9	56	119	23
	Minimum diversity, sample 2	24	61	-4	65	125	130	23	-57
4 ≤ IC ≤ 8	Phyletic profiles	64	85	31	13	46	10	12	
	Random sampling	118	35	-6	44	-13	39	-21	-20
	Maximum diversity sampling	103	71	-3	27	7	15	3	-14
	Minimum diversity, sample 1	96	28	-5	14	30	4	43	15
	Minimum diversity, sample 2	80	21	25	-3	56	40	33	18
IC < 4	Phyletic profiles	175	45	19	14	15	11	-1	
	Random sampling	188	13	10	16	12	5	4	-1
	Maximum diversity sampling	184	28	5	19	6	1	5	6
	Minimum diversity, sample 1	173	16	12	2	5	14	41	24
	Minimum diversity, sample 2	166	14	8	12	14	52	16	26

16S rRNA sequencing

(b) Biological process

IC		≤ 20	≤ 50	≤ 100	≤ 200	≤ 500	≤ 1000	≤ 2071	≤ 5049	≤ 10000	≤ 20570
IC > 8	Phyletic profiles	126	116	130	-4	-72	14	59			
	Random sampling	109	208	-3	29	-28	-60	14	-41	129	-31
	Maximum diversity sampling	135	64	49	-91	89	-71	66	-13	-109	110
	Minimum diversity sampling	73	44	-12	48	-56	11	15	174	98	47
4 ≤ IC ≤ 8	Phyletic profiles	64	176	109	40	40	6	14			
	Random sampling	93	21	53	5	-2	23	8	27	11	12
	Maximum diversity sampling	86	64	43	9	-3	-2	26	23	-7	22
	Minimum diversity sampling	67	12	-3	18	0	15	-4	95	49	69
IC < 4	Phyletic profiles	128	46	26	6	8	9	2			
	Random sampling	139	9	5	8	3	4	-2	3	5	3
	Maximum diversity sampling	138	13	8	9	1	1	3	2	2	5
	Minimum diversity sampling	129	12	3	1	7	2	-2	27	11	14

(b) Molecular function

IC		≤ 20	≤ 50	≤ 100	≤ 200	≤ 500	≤ 1000	≤ 2071	≤ 5049	≤ 10000	≤ 20570
IC > 8	Phyletic profiles	130	116	13	-91	118	-54	56			
	Random sampling	144	26	21	38	14	26	-47	26	-71	67
	Maximum diversity sampling	153	57	-19	-91	-21	102	-51	118	-16	-39
	Minimum diversity sampling	114	14	-70	163	-53	-14	16	97	55	24
4 ≤ IC ≤ 8	Phyletic profiles	55	78	55	38	20	3	14			
	Random sampling	90	8	11	21	3	10	8	2	-17	24
	Maximum diversity sampling	89	11	38	-14	-15	35	-1	-7	20	11
	Minimum diversity sampling	65	32	-2	17	18	-12	-1	61	12	24
IC < 4	Phyletic profiles	141	30	25	23	6	3	5			
	Random sampling	153	7	14	1	3	7	1	3	2	-1
	Maximum diversity sampling	154	7	14	3	-2	11	-3	5	-3	5
	Minimum diversity sampling	143	9	3	11	6	2	0	20	11	14

(b) Cellular component

IC		≤ 20	≤ 50	≤ 100	≤ 200	≤ 500	≤ 1000	≤ 2071	≤ 5049	≤ 10000	≤ 20570
IC > 8	Phyletic profiles	120	76	82	-11	135	-135	24			
	Random sampling	169	-57	65	-39	50	30	-10	-35	75	-22
	Maximum diversity sampling	106	172	-27	108	-76	91	-53	88	-81	35
	Minimum diversity sampling	85	-1	106	39	97	-219	184	46	20	77
4 ≤ IC ≤ 8	Phyletic profiles	73	101	37	15	55	14	13			
	Random sampling	118	6	10	19	-4	21	-23	27	-12	12
	Maximum diversity sampling	120	6	18	9	-2	3	3	11	-4	6
	Minimum diversity sampling	105	19	-20	25	17	7	1	25	-2	32
IC < 4	Phyletic profiles	175	45	19	14	15	11	-1			
	Random sampling	194	3	8	9	2	0	-3	8	-6	6
	Maximum diversity sampling	193	15	3	5	0	0	-1	5	5	3
	Minimum diversity sampling	180	17	-8	13	8	-1	2	30	11	7

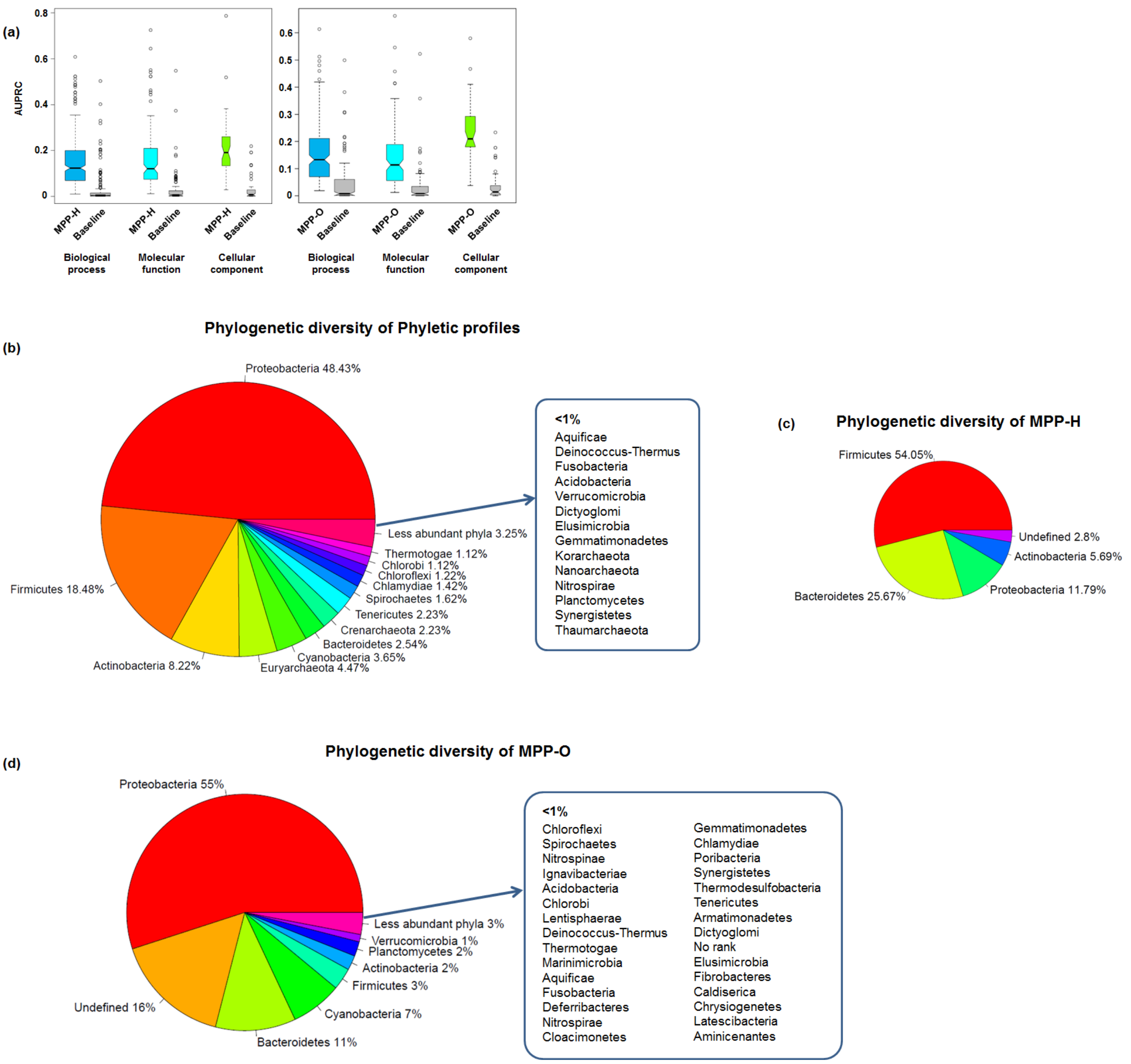

Fig. S1. Predictive accuracy and phylogenetic diversity of the MPP-H and MPP-O data sets. (**a**) Distribution of MPP-H and MPP-O accuracies (expressed as AUPRC) on 451 and 325 learnable GO functions, respectively. GO functions are divided in groups according the GO domain. Baselines are constructed from randomized MPP-H/MPP-O data obtained by randomly assigning GO functions to COGs in the same proportions they were assigned to the original data. (**b**-**d**) Phylogenetic diversity of phyletic profiles composed of 985 microorganisms (**b**), MPP-H (**c**) [1] and MPP-O (**d**) [2], expressed on the level of phyla. Abbreviations: MPP = metagenome phyletic profiles.

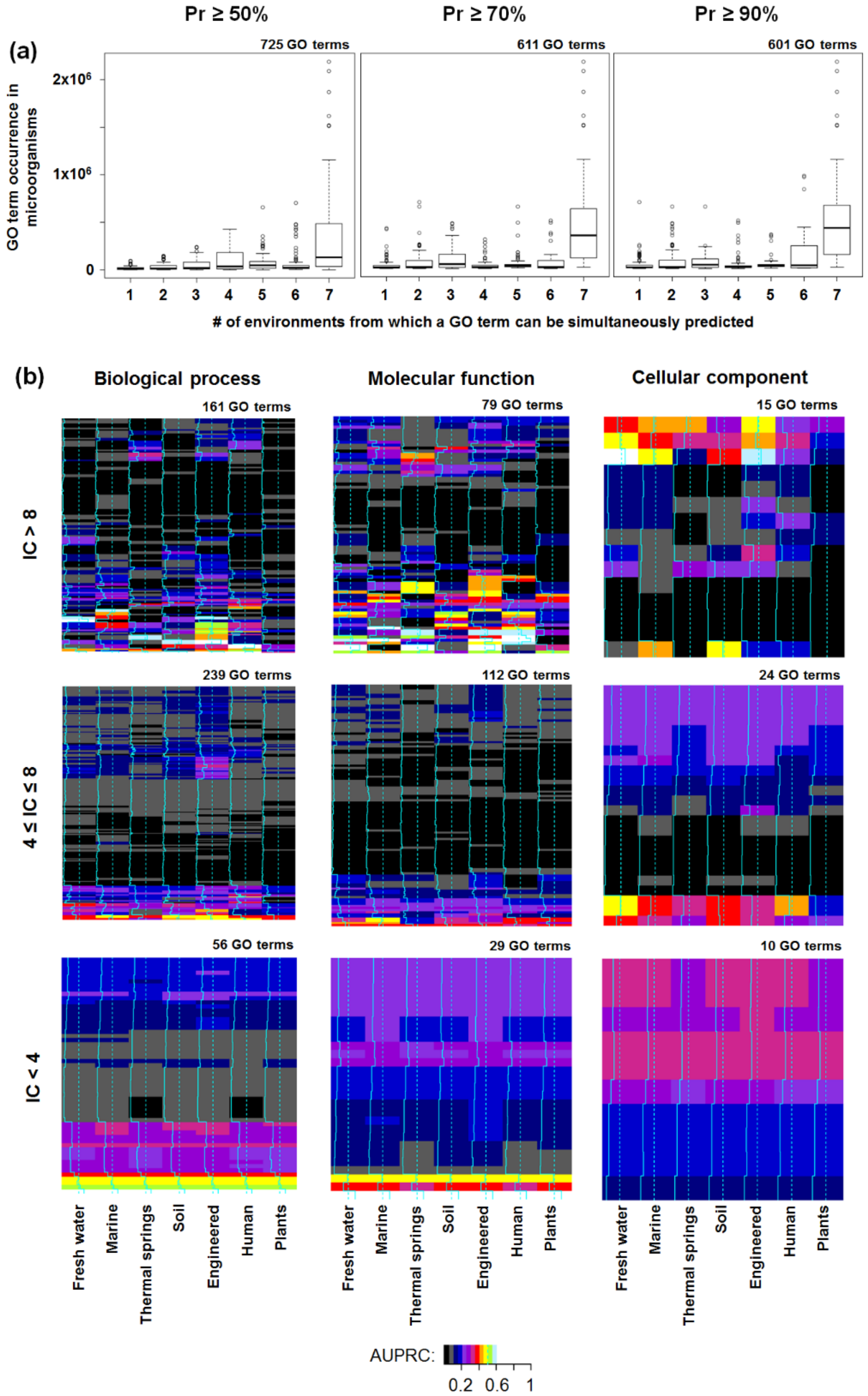

Fig. S2. Model accuracy in predicting GO functions from metagenomes representing distinct environments. (**a**) GO functions that are predicted from all seven environments are associated with COGs that are frequently-occuring in microbial genomes. GO function occurrence (y-axis) is measured as the sum of the number of microorganisms in which each COG having that function occurs. (**b**) Predictive accuracy of GO functions expressed as function-specific accuracy of the environment-representing MPP. Rows in heatmaps represent GO functions, columns environments and brighter colors higher accuracy (expressed as cross-validation AUPRC). The first two heatmaps in the first row are equal to the heatmaps in Fig. 2b. IC stands for information content.

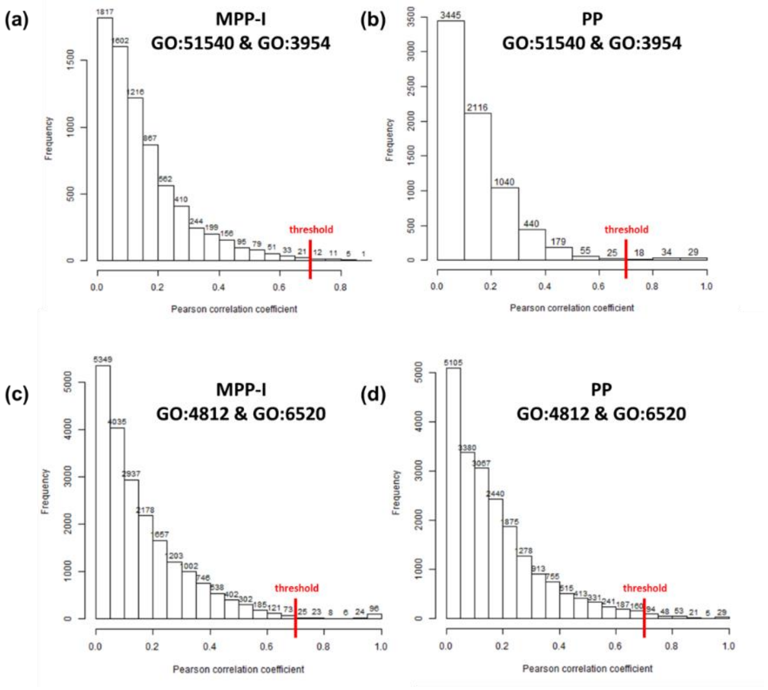

Fig. S3. Distributions of COG pairwise similarities, related to networks in Fig. 3. (**a**-**d**) Histograms represent distributions of non-zero COG similarities (COGs from Fig. 3; similarity is measured using Pearson correlation coefficient; absolute values of the coefficients are considered here) computed from MPP-I or matched PP profiles. Similarities are computed using metagenomes/genomes with positive values of Random Forests feature importance (Gini-based). The threshold of 0.7 represents the point above which edges in the networks were retained. MPP, metagenome phyletic profile. PP, phyletic profile.

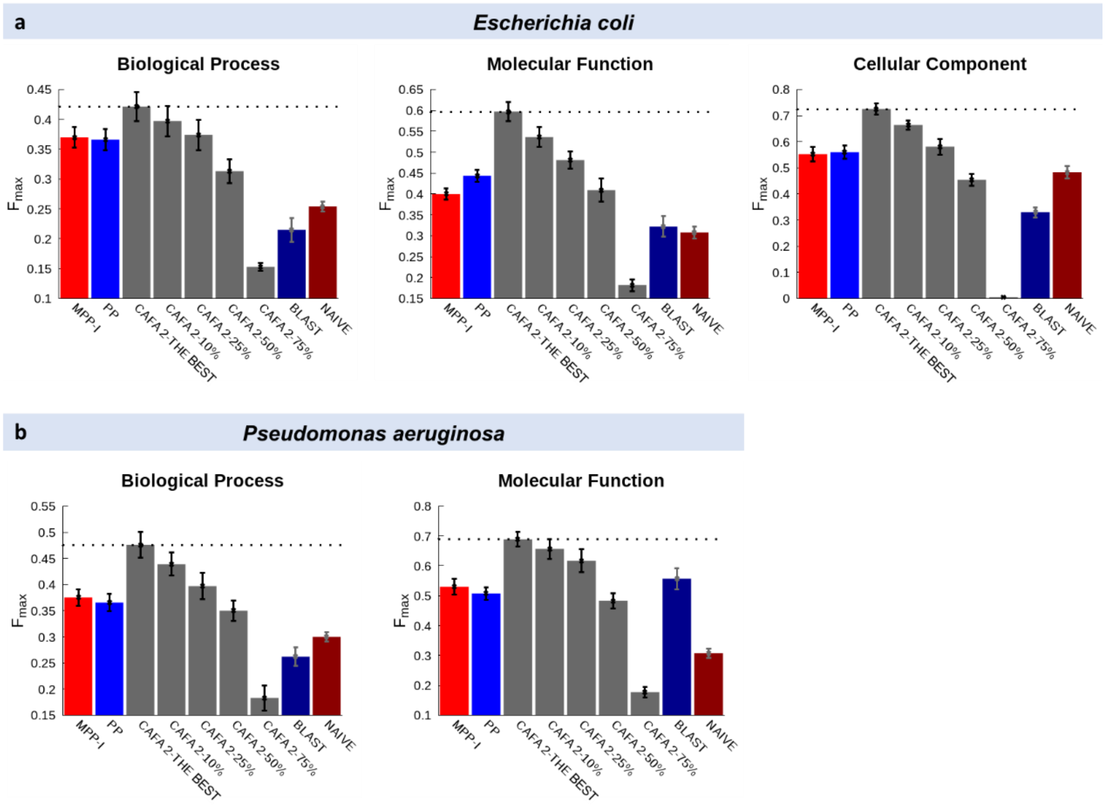

Fig. S4. Performance of PP and MPP-I classifiers on the prokaryotic CAFA 2 validation sets. The F_{max} accuracy measure is determined as in the CAFA 2 publication; error bars are standard deviations, obtained by bootstrapping the set of benchmark genes.

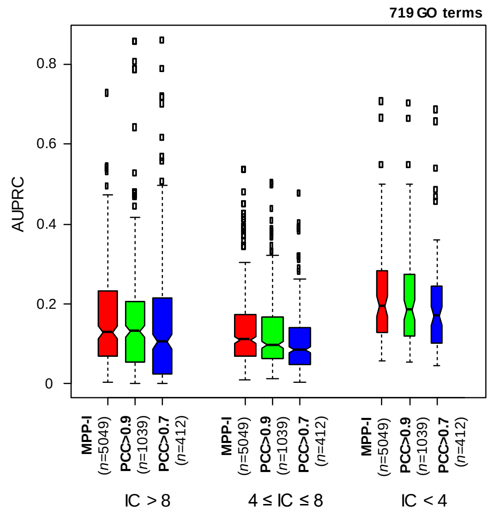

Fig. S5. Removing redundant features is not in itself sufficient to improve accuracy of classifiers based on the MPP-I metagenomic dataset. PCC, Pearson's correlation coefficient (between pairs of metagenomes; n denotes the number of remaining metagenomes in the dataset). IC, information content (of GO terms). AUPRC, area under the precision-recall curve.

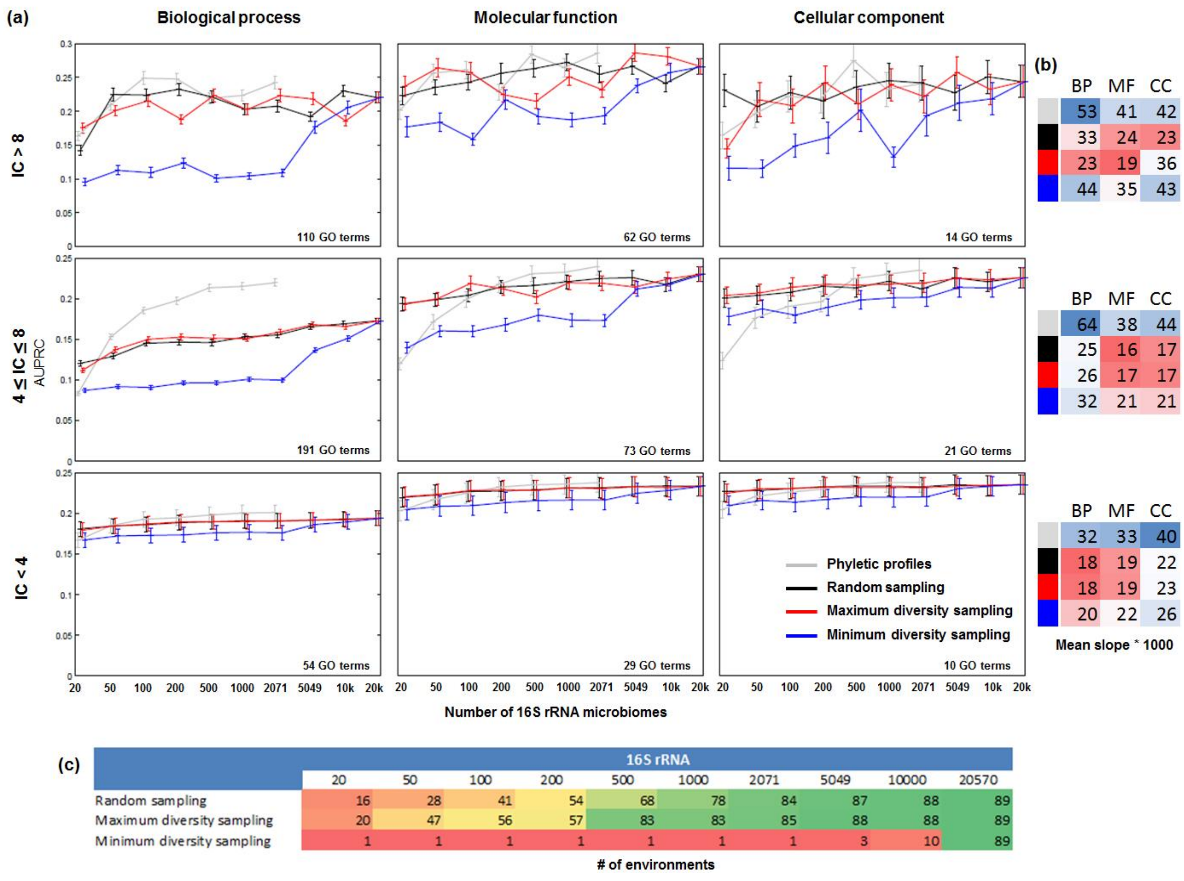

Fig. S6. Effects of diversity and the total number of 16S rRNA data sets on accuracy of gene function prediction. (**a**) X-axes represent the number of sampled microbiomes with 16S rRNA gene sequencing data. Y-axes represent cross-validation AUPRC averaged over GO functions from a specific domain and of a specific level of generality (IC). Error bars represent standard error of the mean. Maximum diversity sampling tends to retain the same ratio of samples from the environments represented in the data set. Minimum diversity sampling always begins with the largest environment. (**b**) represents slopes of the regression lines for phyletic profiles and metagenome phyletic profiles with different sampling approaches, as average over the slopes of segments connecting points in plot; complete table in Table S6b. (**c**) shows the number of environments represented in each data set. Abbreviations: BP = Biological process; MF = Molecular function; CC = Cellular component; IC = Information content.

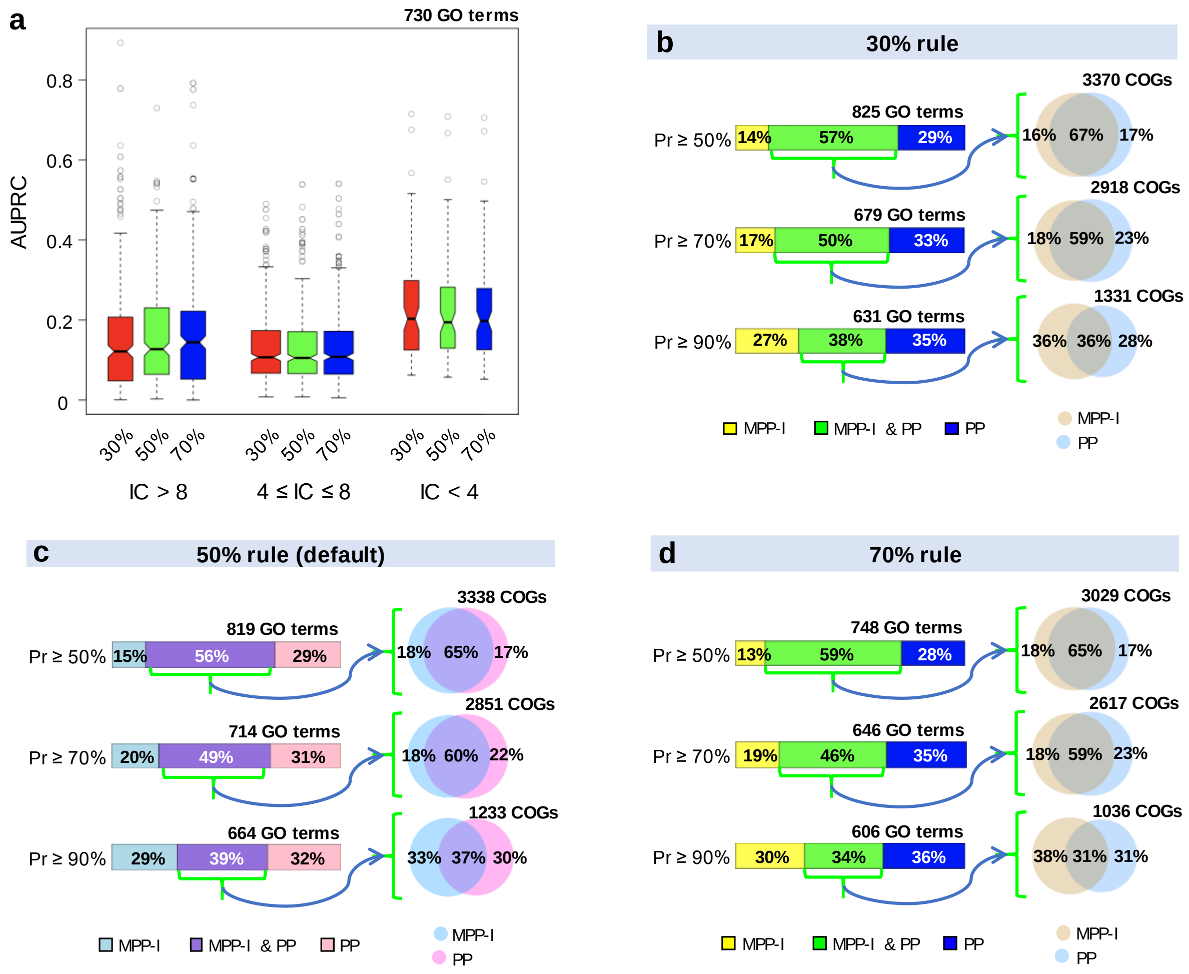

Fig. S7. Varying the stringency of rule for GO term propagation within gene families. Changing the "≥50% genes" heuristic for propagating gene function within a COG/NOG towards higher strigency (≥70%) or lower stringency (≥30%) has only minor effects on accuracy (**a**) and complementarity of predictions provided by PP and MPP-I classifiers (**b**-**d**). Explanation of diagrams in legend of Fig. S8.

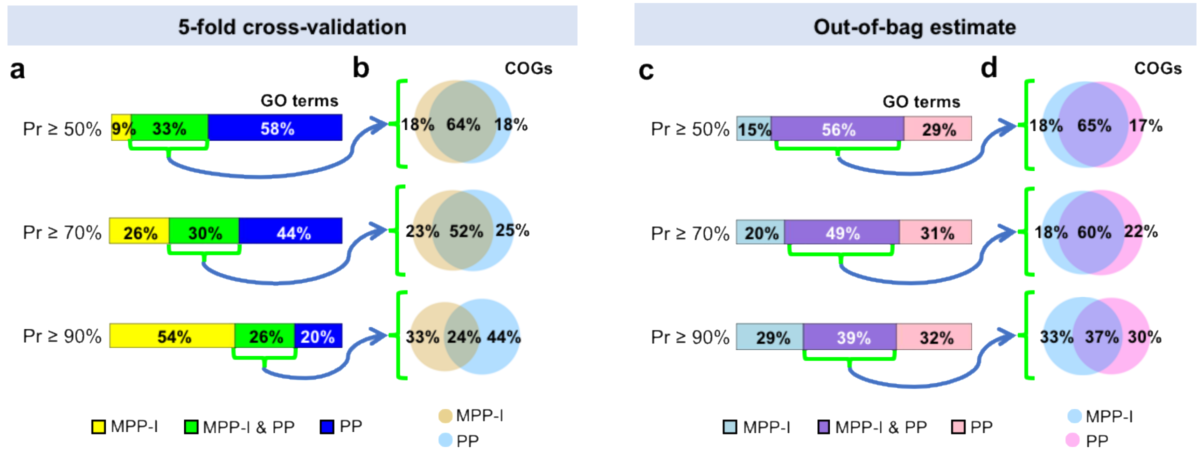

Fig. S8. Similar complementarity patterns using out-of-bag estimation and 5-fold cross-validation. Overlap between MPP-I and matched PP in terms of: (**a** and **c**) percentages of GO functions that can be predicted at different levels of precision only by MPP-I, only by PP or by both; (**b** and **d**) percentages of COG gene families to which only MPP-I, only PP or both can assign GO functions (considering those GO functions that can be simultaneously predicted by both MPP-I and PP, represented by the middle part of the bars in **a** and **c**).

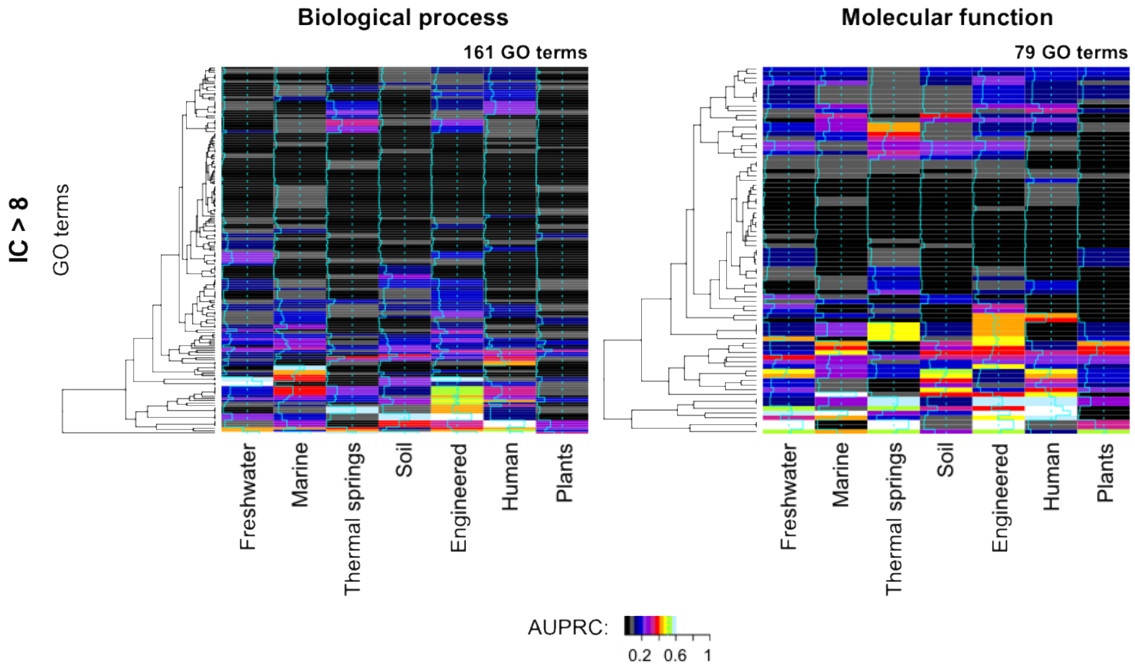

Fig. S9. Accuracy of classification models resulting from different environments. Same data as Fig. 2b, but with dendrograms showing the hierarchical clustering of the rows, containing classifier accuracy (as AUPRC score) in predicting various GO terms across the seven environments present in the MPP-I data set.

References for Additional file 1.

[1] Li, Y., Calvo, S. E., Gutman, R., Liu, J. S., & Mootha, V. K. Expansion of biological pathways based on evolutionary inference. *Cell* **158**, 213-225 (2014).

[2] Sunagawa, S. *et al.* Structure and function of the global ocean microbiome. *Science* **348**, 1261359 (2015).
